# The effect of developmental stages on microbiome assembly in the phyllosphere and rhizosphere of rice grown in urban area soil

**DOI:** 10.1186/s40793-025-00748-9

**Published:** 2025-07-11

**Authors:** Qianze Peng, Shu’e Sun, Jiejia Ma, Silu Chen, Liming Gao, Xiaohua Du, Xian Liu, Feiying Zhu, Weiye Peng, Yong Liu, Pin Su, Tomislav Cernava, Deyong Zhang

**Affiliations:** 1 School of Tropical Agriculture and Forestry, Hainan University, Haikou, 570228 China; 2 State Key Laboratory of Hybrid Rice and Institute of Plant Protection, Hunan Academy of Agricultural Sciences, Changsha, 410125 China; 3 National Center of Technology Innovation for Saline-Alkali Tolerant Rice in Sanya City, Sanya, 572024 China; 4 Longping Branch, College of Biology, Hunan University, Changsha, 410082 China; 5 College of Orient Science and Technology, Hunan Agricultural University, Changsha, 410128 China; 6 School of Biological Sciences, Faculty of Environmental and Life Sciences, University of Southampton, Southampton, SO17 1BJ UK

**Keywords:** Phyllosphere, Rhizosphere, Urbanization, Rice, Microbiome assembly, Plant Microbiome

## Abstract

**Background:**

The plant microbiome can support plant health and fitness in the face of biotic and abiotic stress. Research has mostly focused on plant growth in natural and agricultural soils. However, as urban areas continue to expand and soils change in the Anthropocene, microbiome assembly during development of plants grown in urban area soil remains largely elusive. Here, we examined the effect of developmental stages on the phyllosphere and rhizosphere microbiomes of rice grown in soil from an urban area during the vegetative growth stages.

**Results:**

We found that the microbial alpha and beta diversity, networks, and functions of the phyllosphere and rhizosphere microbiomes significantly differed among rice seedling, tillering, and elongation stages. Notably, we observed that bacteria assigned to potential animal parasites or symbionts not only exhibited significantly higher relative abundances in the phyllosphere compared to the rhizosphere but are also influenced by the developmental stages. Plants grown in the urban area soil had a higher relative abundance of Bacteroidales and enriched bacteria assigned to potential animal parasites or symbionts in the phyllosphere in contrast to plants grown in field. Some of these bacteria were shown to significantly influence the assembly of the phyllosphere microbiome and to prevalently engage in negative interactions with other microbes.

**Conclusion:**

Our study provides new insights into developmental stage-resolved microbiome assembly of plants grown in urban areas. The insights could help in the development of strategies for promoting ‘*One Health*’ by highlighting the role of plants as alternative host for bacterial groups that are prevalently associated with animals.

**Supplementary Information:**

The online version contains supplementary material available at 10.1186/s40793-025-00748-9.

## Introduction

The plant microbiota comprises beneficial, neutral and pathogenic microorganisms, including bacteria, fungi, protists, nematodes and viruses [1, 2]. Microbial communities, which inhabit the rhizosphere (root-soil interface), phyllosphere (all the aboveground compartments of plants, including the leaf, flower, stem, and fruit) and endosphere (internal tissues), have been shown to promote plant growth, nutrient uptake and pathogen resistance [3–5]. Known sources of the plant microbiota include soil, seeds, dust, air, rainwater, and animal vectors [6]. In addition, the plant microbiome is shaped by complex interactions among the host, microbes and the environment [7, 8]. Previous studies have shown that many environmental and ecological factors, such as climate, pathogen infection, anthropogenic activities and soil type can substantially influence assembly of the microbiota [9–11]. Within the holobiont (multicellular host and its associated microbiota as a functional entity), plant growth, immune response and genotype, microbe-microbe interaction, and metabolites together contribute to the dynamics observed in such communities [12–15]. Previous research on plant microbiome assembly has mainly focused on the impacts of the host, other microbes, and the environment.

Soils are a cornerstone of ‘*One Health*’, and serve as a source and reservoir of pathogens, beneficial microorganisms and the overall microbial diversity, which determines plant, animal and human microbiomes [16]. They can have direct and indirect influences on soil, plant, animal, and human health [17, 18]. Notably, urban greenspaces were shown to increase the diversity of human gut and skin microbiota and thus enhance immune regulation [19, 20]. Plant microbiome research has mostly focused on plant growth in natural and agricultural soils [21]. Recent observations have shown that soil microbiomes in urban areas, compared with rural areas, contain more antibiotic resistance genes and genes associated with human pathogens [22]. Land-use intensification, urbanization, and landscape simplification cause homogenization of the soil microbiome, reducing soil microbial diversity and causing variations in the plant microbiome [11]. Urban greenspaces were also shown to have a higher proportion of plant and human pathogens in the soil [23]. Aboveground plant compartments play a crucial role in shaping the airborne microbiome, including the exchange of pathogens within the air-phyllosphere-soil continuum in urban greenspaces [24]. Human-driven disturbances and industrial development lead to an intensification of biotic and abiotic stresses, each of which influences microbial community assembly, microbial functional traits, and the antibiotic resistome of soil and phyllosphere microbiomes in urban green spaces [25–28]. Moreover, bacterial communities are the most susceptible to change across urban environments compared with fungal and protozoan communities [29]. Although there have been extensive studies on how anthropogenic activities affect microbiome assembly of plants grown in urban green spaces, the role of urban soil in plant microbiome assembly remains elusive.

Recent studies have also highlighted the significant contribution of plant developmental stages on plant microbiome assembly [9, 30]. This is expected given that the plant morphology, exudates and immune response, which differ among developmental stages, are among the specific traits by which plants modulate their microbial communities [30]. In field experiments, the microbiomes of rice [31, 32], maize [33], grapevine [34, 35] and strawberry [36] were shown to be shaped by developmental stages. These studies focused on microbial community dynamics of plants grown in field soil. One of our recent studies indicated that rice and maize growth in urban areas selectively assemble microbiomes from soil [37]. However, microbiome assembly during different development stages of plants grown in urban areas is still not well understood.

In this study, rice was planted in soil obtained from an urban area located at traffic intersections to enable the plants to acquire their microbiome from an alternative source. Plants were cultivated in pots in a glasshouse to minimize the influence of other environmental factors. Previous research has shown that the rice microbiome tends to stabilize from the beginning of the reproductive stage [31, 32]. Therefore, this study only focused on the phyllosphere and rhizosphere microbiomes during the seedling, tillering, and elongation stages of the rice vegetative growth. We also compared the microbiome of the phyllosphere of field-grown rice and the urban area soil. Additionally, we specifically explored the effects of potential animal parasites or symbionts on plant microbiome assembly. Our results provide insights into implications of host development in rice microbiome assembly in urban areas.

## Materials and methods

### Data collection, plant material, seed germination, and pot experiments

For comparative assessments, publicly available microbiome datasets obtained from urban and field samples were obtained from NCBI (BioProject: PRJNA1158963 [38], and PRJNA919006 [39]), CNCB (PRJCA016320 [13], and PRJCA001214 [40]) (Supplementary Table 1).

Seeds of the rice variety ZH11 used for experiments in this study were obtained from the Hunan Hybrid Rice Research Center (HHRRC, Changsha, China). Plants were cultured under relative humidity kept at 80% and 28 °C in a glasshouse from July to August. All rice plants were grown in pots that that contained soil from the urban area. It was collected from a traffic intersection in Changsha (28°11′44.963″ N, 113°05′1.684″ E), Hunan Province, China (Fig. S1). The top 10–20 cm of soil was collected and sieved (3-mm sieve) to remove rocks and other debris and dried at room temperature. Then, the soil was mixed with sterile water (1:1 w/v), followed by supplementation with sterile half-strength Murashige and Skoog (MS) medium solution (pH 5.8) (1:1 w/v). Rice seeds were surface-sterilized using 75% ethanol (EtOH) for 2 min and 7% sodium hypochlorite (NaOCl) supplemented with 0.2% Triton X-100 three times for 8 min, following washing with sterile water six times [13]. Sterile seeds were then immersed in sterile water for 1 d at 4 °C in the dark. Subsequently, all rice seeds were sowed into the pots prepared with the aforementioned soil mixture. Each pot was watered with sterile water once a week and sterile half-strength MS solution once a week after germination. The water was autoclaved at 121 °C for 20 min to ensure sterility.

### Sample collection

The samples were enriched for their bacterial fraction based on previous studies [41, 42]. Leaf and root samples were collected at seedling, tillering and elongation stages (21, 42, 63 days after sowing); all samples were collected after three days of application of sterile water and half-strength MS. The leaves and roots were removed using sterile scissors and rubber gloves (surface-sterilized using 75% EtOH and washed with sterile water six times for each plant) for collection of phyllosphere and rhizosphere bacteria, respectively (six replicates per compartment, each replicate contained three plants).

Rice roots were washed with sterile water to remove loosely attached soil particles, retaining only 1–2 mm of root-attached soil for rhizosphere sampling. Then, a total of 1 g leaf and root material from each replicate were used to enrich the microbiota from the plant phyllosphere and rhizosphere [13, 37]. The leaf and root samples for each replicate were transferred into a 50-ml plastic tube containing 20 ml sterile PBS (phosphate buffered solution; 0.02 M, PH = 7.0). The tube was placed in a shaker (Yiheng, Shanghai, China) for 10 min set at 200 rpm/min and then sonicated for 5 min at a frequency of 30 kHZ at 4 °C in a ultrasonic cleaner (Yu clean, Shenzhen, China). After collection of the suspensions, the leaves and roots were treated with the same oscillation and sonication procedures twice to ensure that bacterial cells were thoroughly washed off from the leaf and root surface. The suspensions were pooled together and subjected to centrifugation (16,980 g, 10 min, 4 °C). After combining all suspensions, the samples were stored at -80 °C before further use.

### DNA extraction, PCR amplification, and sequencing

The DNA extraction and PCR amplification were based on a previous study [13]. Total DNA was extracted using the MagPure Soil DNA LQ Kit (Magen, Shanghai, China) according to the manufacturer’s instructions. Quality and quantity of DNA was verified using a NanoDrop ND-1000 spectrophotometer (Thermo Fisher Scientific, USA) and agarose gel electrophoresis, respectively. Extracted DNA was diluted to a concentration of 1 ng/µl and stored at -20°C until further processing. PCR amplification of bacterial 16S rRNA gene fragments (V3-V4 region) was performed using Takara Ex Taq polymerase mix (Takara, Beijing, China) and the primers 343F (5’-TACGGRAGGCAGCAG-3’) and 798R (5’-AGGGTATCTAATCCT-3’) [13]. Amplification was confirmed using agarose gel electrophoresis and the products purified using Agencourt AMPure XP beads (Beckman Coulter, Pasadena, USA) twice. After purification, the DNA was quantified using the Qubit dsDNA assay kit (High Sensitivity, 0.2–100 ng/µl; Yeasen, Shanghai, China). Equal amounts of purified DNA were pooled for sequencing on the NovaSeq 6000 platform (Illumina Inc, USA) at Shanghai OEbiotech (Shanghai, China).

### Bioinformatic analysis for phyllosphere and rhizosphere Microbiome profiling

Bioinformatic analyses for phyllosphere and rhizosphere microbiome profiling were based on a previous study [13, 40]. The 16S rRNA gene fragment sequences were processed using vsearch v.2.22.1 [43]. Paired-end reads were merged, low-quality sequences were filtered, and the primers were removed using ‘fastq_mergepairs’, ‘fastx_filter’, and ‘fastq_stripleft’ commands in vsearch, respectively. After trimming, paired-end reads were dereplicated, clustered and denoised using ‘derep_fulllength’, ‘cluster_size’, and ‘uchime_ref’ commands in vsearch, respectively. Unique reads were clustered into OTUs with 97% similarity. An operational taxonomic unit (OTU) table was generated using ‘usearch_global’ command in vsearch (Supplementary Data 1). All OTUs were annotated using the Silva v138.1 reference database [44]. Bacterial functional profiles were predicted using functional annotation of prokaryotic taxa (FAPROTAX) [45]. Potential animal parasites or symbionts (APS) were identified and their taxonomy assigned with FAPROTAX (Supplementary Data 1).

Alpha diversity analysis was carried out using the EasyAmplicon script [46]. Rarefaction analysis confirmed that the implemented approach sufficiently captured microbial diversity in the rhizosphere and phyllosphere of all investigated groups. (Fig. S2). A unconstrained and constrained principal coordinate analysis (PCoA and CPCoA) based on Bray-Curtis distances was performed using the R package vegan [47]. Differences in beta diversity were assessed using a permutational analysis of variance (PERMANOVA, 999 permutations) available in the R package vegan. Analysis of differential species and functions abundance and determination of significantly different abundant taxa and functions was performed using an unpaired two-tailed Student’s t-test. Random forest analysis was conducted to identify the important predictors based on the mean decrease in Gini coefficient (MDG), which indicated the variable contributes to the homogeneity of the nodes and leaves in the resulting random forest for different developmental stages using the “randomForest” R package [48], and evaluation of model performance used nested cross-validation (10-fold inner loop, 5-fold outer loop) based on a previous study [31]. Redundancy analysis (RDA) was performed using the R package vegan. Differences in the effect size of the microbiome were assessed using a permutation test (999 permutations) with the ‘envfit’ command in the R package vegan. The relevant data were visualized by using the R package ggplot2 [49].

Microbial co-occurrence network analysis of bacterial OTUs was performed using the R packages NetCoMi [50] and igraph [51] based on the SparCC method. Visualization of the microbial networks and estimation of node-level topological features (degree, betweenness centrality, closeness centrality and eigen vector centrality) and network-level topological features (average degree and clustering coefficient) for each developmental stage were performed by using Gephi (v0.9.2; https://gephi.org) [52]. OTUs belonging to the highest scoring in terms of node degree and Eigen vector centrality in the network were identified as potential hub OTUs [53]. Scripts used for all analyses described here were made publicly available on Github [https://github.com/PengQianze/microbiome-analysis-code.git].

## Results

### Phyllosphere and rhizosphere Microbiome features of rice grown in soil from an urban area

**Fig. 1:**
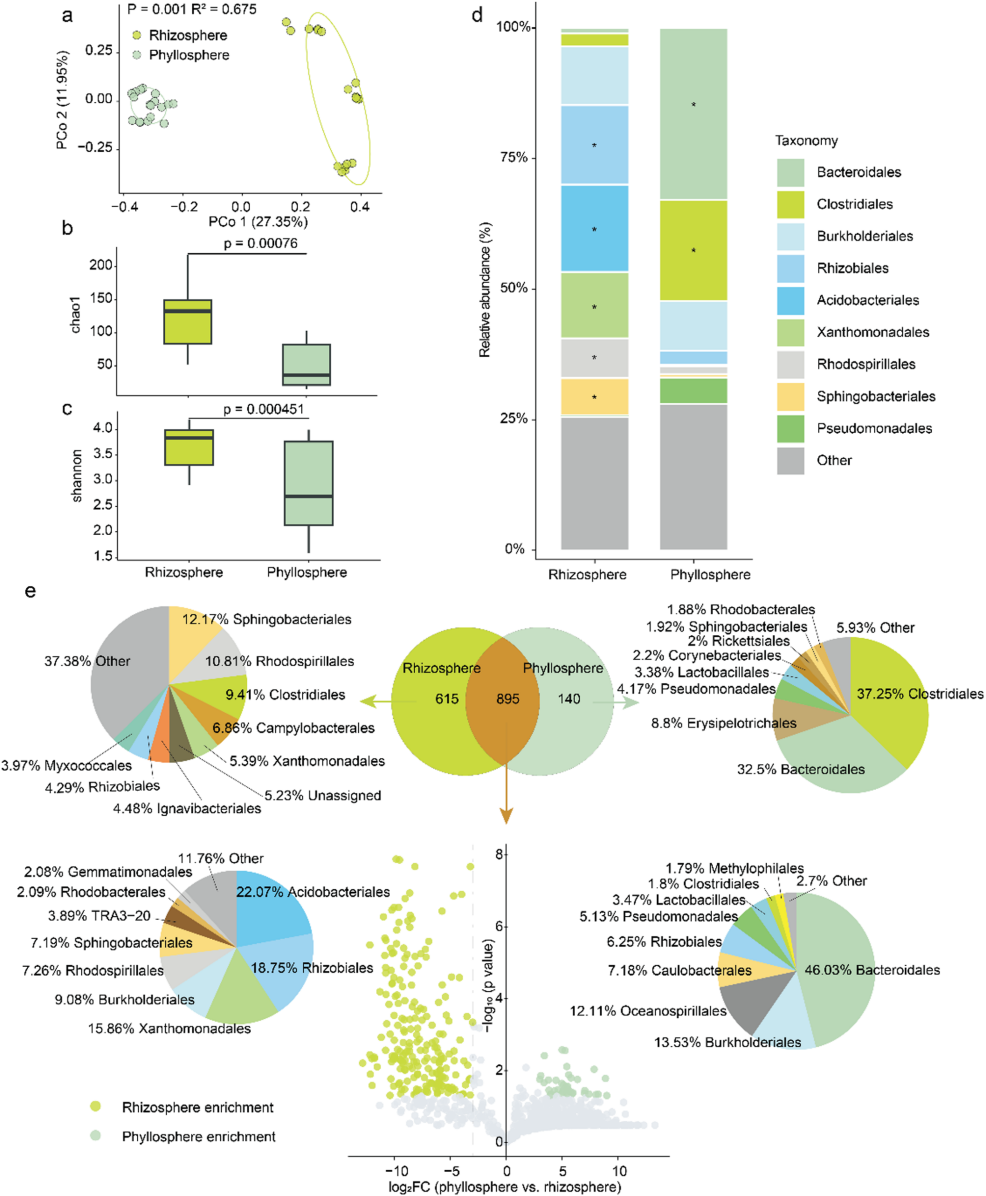
Microbiome comparisons for the phyllosphere and rhizosphere of rice. **(a**) Unconstrained PCoA (for principal coordinates PCo1 and PCo2) based on Bray-Curtis distances showing bacterial community clustering in the phyllosphere and rhizosphere (p value was calculated by one-way PERMANOVA). Ellipses cover 68% of the data for each habitat. Chao1 (**b**) and Shannon index (**c**) for bacterial communities inhabiting the phyllosphere and rhizosphere. The horizontal bars within boxes represent medians. The tops and bottoms of boxes represent the 75th and 25th percentiles, respectively. The upper and lower whiskers extend to data no more than 1.5× the interquartile range from the upper edge and lower edge of the box, respectively. **(d)**, Order-level distribution of bacteria in the rhizosphere and phyllosphere microbiomes. Asterisks represent significant differences between rhizosphere and phyllosphere as assessed with an unpaired t test (*p* < 0.05). (**e**) OTU distribution in the rhizosphere and phyllosphere microbiomes. The Venn diagram in the middle represents the distribution of OTUs in the rhizosphere and phyllosphere. The pie chart above represents the order-level composition of unique OTUs in the rhizosphere and phyllosphere. The volcano plot indicates OTUs that were significantly enriched in the rhizosphere and phyllosphere (*p* < 0.05,| log_2_(Fold change)| >3). The pie chart below illustrates the order-level composition of OTUs significantly enriched in the rhizosphere and phyllosphere. The replications of phyllosphere and rhizosphere samples are 18. In this figure, the p value was calculated with unpaired two sides t-test

Many studies have shown that plant compartments have a significant impact on the diversity and composition of the microbiome [33, 54, 55]. Here, we also found significant differences in the microbiome between the phyllosphere and rhizosphere of rice grown in urban area soil. Bacterial communities obtained from the phyllosphere and rhizosphere formed two distinct clusters, as indicated by the unconstrained principal coordinate analysis (PCoA) of Bray-Curtis distances (Fig. 1a; *p* = 0.001, R^2^ = 0.675, PERMANOVA). This observation suggested that the rhizosphere and phyllosphere had distinct microbiome compositions and structures. Further analysis revealed that Chao1 richness (Fig. 1b) and Shannon index (Fig. 1c) of the rhizosphere bacterial community were higher than those of the phyllopshere community, suggesting that the rice rhizosphere microbiome was more diverse.

The predominant bacterial orders of both the rhizosphere and phyllosphere were comprised of Bacteroidales (32.9% and 1% of relative abundance for phyllosphere and rhizosphere, respectively), followed by Clostridiales (19.3% and 2.5%), Burkholderiales (9.6% and 11.2%), Rhizobiales (2.7% and 15.2%), Acidobacteriales (0.2% and 16.7%), Xanthomonadales (0.2% and 12.7%), Rhodospirillales (1.4% and 7.6%), Sphingobacteriales (0.7% and 7.1%), and Pseudomonadales (5% and 0.3%) (Fig. 1d). Compared to the phyllosphere, the rhizosphere had a higher relative abundance of Rhizobiales, Acidobacteriales, Xanthomonadales, Rhodospirillales, and Sphingobacteriales, while the phyllosphere had a higher relative abundance of Bacteroidales and Clostridiales (*p* < 0.05) (Fig. 1d, Supplementary Table 2).

Furthermore, a detailed analysis of OTUs revealed distinct microbial patterns in the phyllosphere (140 unique OTUs) and rhizosphere (515 unique OTUs) (Fig. 1e). The dominant abundant bacterial orders of unique OTUs in the phyllosphere were Clostridiales (37.25%), Erysipelotrichales (32.5%), and Bacteroidales (8.8%), while the ones of the rhizosphere were Sphingobacteriales (12.7%), Rhodospirillales (10.81%), and Clostridiales (9.41%) (Fig. 1e). Notably, 895 OTUs (54.24% fo total OTUs) were shared between the phyllosphere and rhizosphere (Fig. 1e). Further differential analysis identified 35 OTUs enriched in the phyllosphere, dominated by Bacteroidales (46%), Burkholderiales (13.5%), and Oceanospirillales (12%) as well as 219 OTUs significantly enriched in the rhizosphere, mainly Acidobacteriales (22.1%), Rhizobiales (18.7%), and Xanthomonadales (15.9%) (Fig. 1e, Supplementary Data 2; *p* < 0.05,| log_2_(Fold change)| >3). These findings underscore the distinct microbial colonization patterns in the phyllosphere and rhizosphere of rice grown in soil from the urban area.

### Developmental stages differently effect Microbiome assembly in the phyllosphere and rhizosphere of rice grown in urban area

**Fig. 2:**
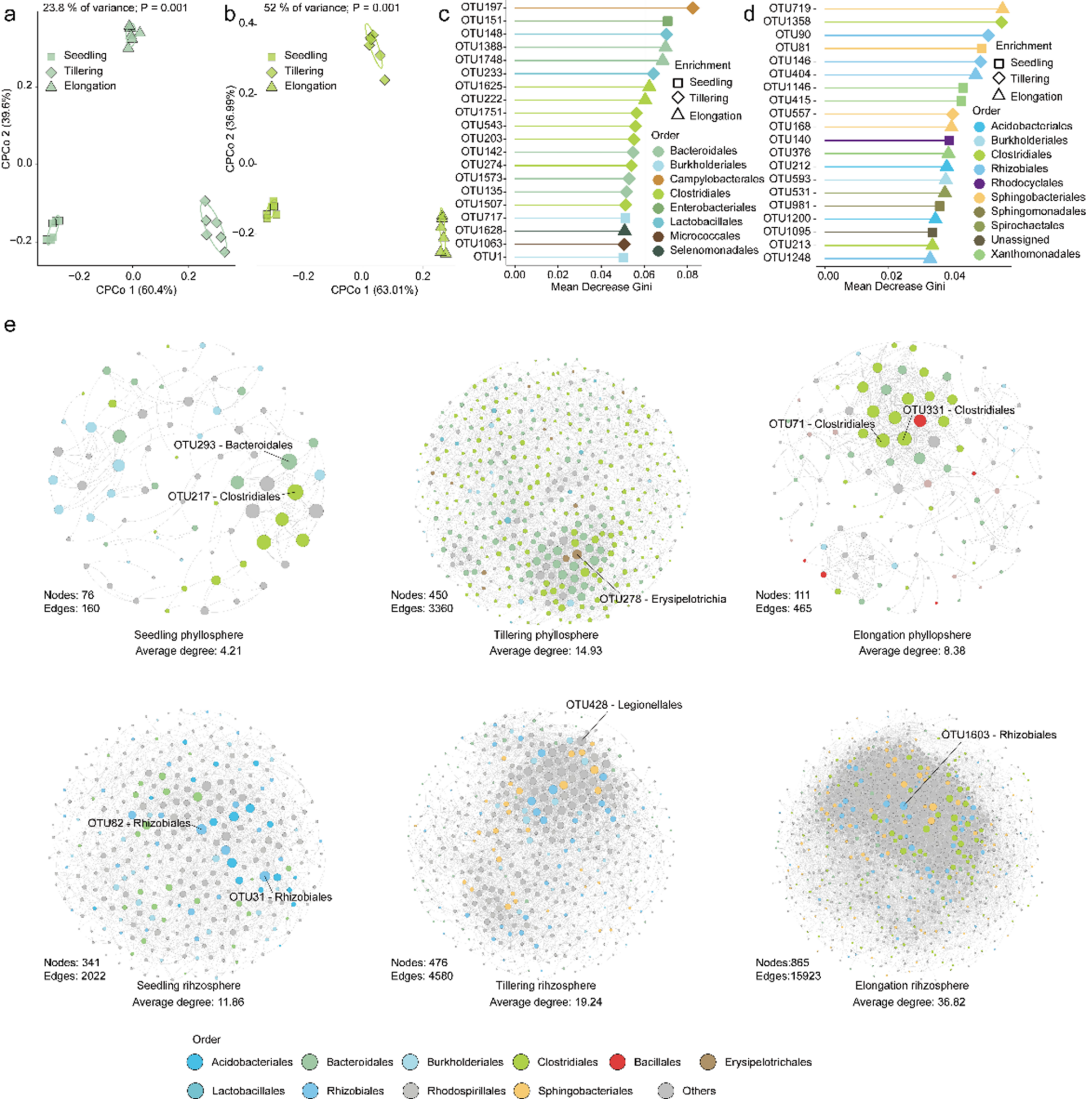
The effects of host developmental stages on the microbiome. Constrained PCoA based on Bray-Curtis distances showing bacterial community separation for seedling, tillering, and elongation developmental stages for (**a**) phyllosphere and (**b**) rhizosphere, respectively (p values were calculated with PERMANOVA). Microbiome members (defined at OTU level) can distinguish developmental stages for (**c**) the phyllosphere and (**d**) the rhizosphere. Important features were identified based on Mean Decrease Gini (MDG) of random forest models. Different shapes represent their enrichment at specific developmental stages. Different colors indicate the order to which this OTU belongs. (**e**) Cooccurrence network analysis showing microbial network patterns for the seedling stage, tillering stage, and elongation stage in the phyllosphere and rhizosphere. Different colors indicate bacterial orders to which the respective OTUs were assigned

To assess the impact of plant developmental stages on the rhizosphere and phyllosphere microbiome of rice grown in soil from the urban area, PERMANOVA analysis and CPCOA ordinations were implemented. Plant developmental stages explained 23.8% of the variation in bacterial communities from the plant phyllosphere (Fig. 2a). Similarly, plant developmental stage explained 52% of the variation for bacterial communities from the plant rhizosphere (Fig. 2b). Moreover, the alpha diversity analysis showed that Chao1 richness (*p* < 0.05) and Shannon index (*p* < 0.05) of bacterial communities were higher during the tillering stage than at the seedling and elongation stages in the phyllosphere (Supplementary Table 3). However, the Chao1 richness (*p* < 0.05) and Shannon index (*p* < 0.05) at the seedling stage were lower than those of the tillering and elongation stages in the rhizosphere (Supplementary Table 4). This observation suggested that plant developmental stages have a differing impact on diversity within the rhizosphere and phyllosphere microbiome of rice grown in urban areas.

To further confirm the robustness of these observations across developmental stages, Random Forest supervised learning models [56] were employed to classify samples and identify which taxa can explain variations. Specifically, OTUs belonging to the bacterial orders Bacteroidales, Burkholderiales, Campylobacterales, Clostridiales, Enterobacteriales, Lactobacillales, Micrococcales, and Selenomonadales were identified as top features in the classification models for developmental stages of the rice phyllosphere (Fig. 2c). For instance, 4 OTUs from the orders Burkholderiales (2 OTUs), Enterobacteriales and Clostridiales were enriched at the seedling stage (Fig. 2c). Twelve OTUs from Clostridiales (5 OTUs), Bacteroidales (3 OTUs), Lactobacillales (2 OTUs), Campylobacterales, and Micrococcales were enriched at the tillering stage (Fig. 2c). Five OTUs from Clostridiales (2 OTUs), Bacteroidales (2 OTUs) and Selenomonadales were enriched at the elongation stage (Fig. 2c). Interestingly, when the random forest (RF) model reached the optimal number of features, the cross-validation error of the RF model was 0.175 in the phyllosphere, while in the rhizosphere, the cross-validation error of the RF model was 0.033 (Fig. S3). The top features in the classification models showed a distinct difference to the phyllosphere. In detail, 5 OTUs from the orders Sphingobacteriales (2 OTUs), Xanthomonadales (2 OTUs), and Rhodocyclales along with an unassigned OTU were enriched at the seedling stage (Fig. 2d). While 4 OTUs from the orders Rhizobiales (2 OTUs), Clostridiales, and Sphingobacteriales were enriched at tillering stage (Fig. 2d). The majority of the OTUs from Acidobacteriales (2 OTUs), Rhizobiales (2 OTUs), Sphingobacteriales (2 OTUs), Burkholderiales, Clostridiales, Spirochaetales, and Xanthomonadales were enriched at the elongation stage (Fig. 2d). These results confirmed that plant developmental stages differently influenced microbiome composition in the phyllosphere and rhizosphere of rice grown in the urban area soil.

We further performed co-occurrence network analysis to assess the impact of developmental stages on phyllosphere and rhizosphere microbiome interactions. Our results showed that microbial network patterns shifted clearly during the three developmental stages and plant compartments (Fig. 2e). Specifically, bacterial taxa had higher network connectivity (average degree = 14.93) at the tillering stage when compared to other stages in the phyllosphere, while this pattern was observed for the elongation stage (average degree = 36.82) in rhizosphere (Fig. 2e). In the microbial network of the phyllosphere, 76, 450 and 111 nodes were obtained for the seedling, tillering and elongation stage, respectively (Fig. 2e). Moreover, the nodes belonged to the bacterial orders Clostridiales, Bacteroidales and Burkholderiales, which were the dominant taxa both at seedling, tillering and elongation stages network in phyllosphere (Fig. S4). In addition, 341, 476, and 865 nodes were obtained at the seedling, tillering and elongation stage in rhizosphere microbial network, respectively (Fig. 2e). The bacterial orders of Rhodospirillales, and Rhizobiales were the dominant taxa both at the networks of seedling, tillering and elongation stages in the rhizosphere (Fig. S5). We further defined the “network hubs” as nodes with highest node values and Eigen vector centrality in the network. nine potential hubs were identified across the three developmental stages in the networks of the phyllosphere and rhizosphere (Fig. 2e). Of those potential hubs in phyllosphere, two belonging to the bacterial orders of Clostridiales and Bacteroidales were identified at the seedling stage. One belonging to the bacterial orders of Erysipelotrichia were identified at the tillering stage (Fig. 2e). And two belonging to the bacterial orders of Clostridiales were identified at the elongation stage (Fig. 2e). In the rhizosphere, two belonging to the bacterial orders of Rhizobiales were identified at the seedling stage (Fig. 2e). And two belonging to the bacterial orders of Legionellales and Rhizobiales were identified at the tillering and elongation stage, respectively (Fig. 2e). Together these results indicated that the diversity, composition and interactions within the microbiota of rice grown in soil of the urban area were influenced by the developmental stages both in the phyllosphere and rhizosphere.

### Functional profiles of phyllosphere and rhizosphere microbiomes

**Fig. 3:**
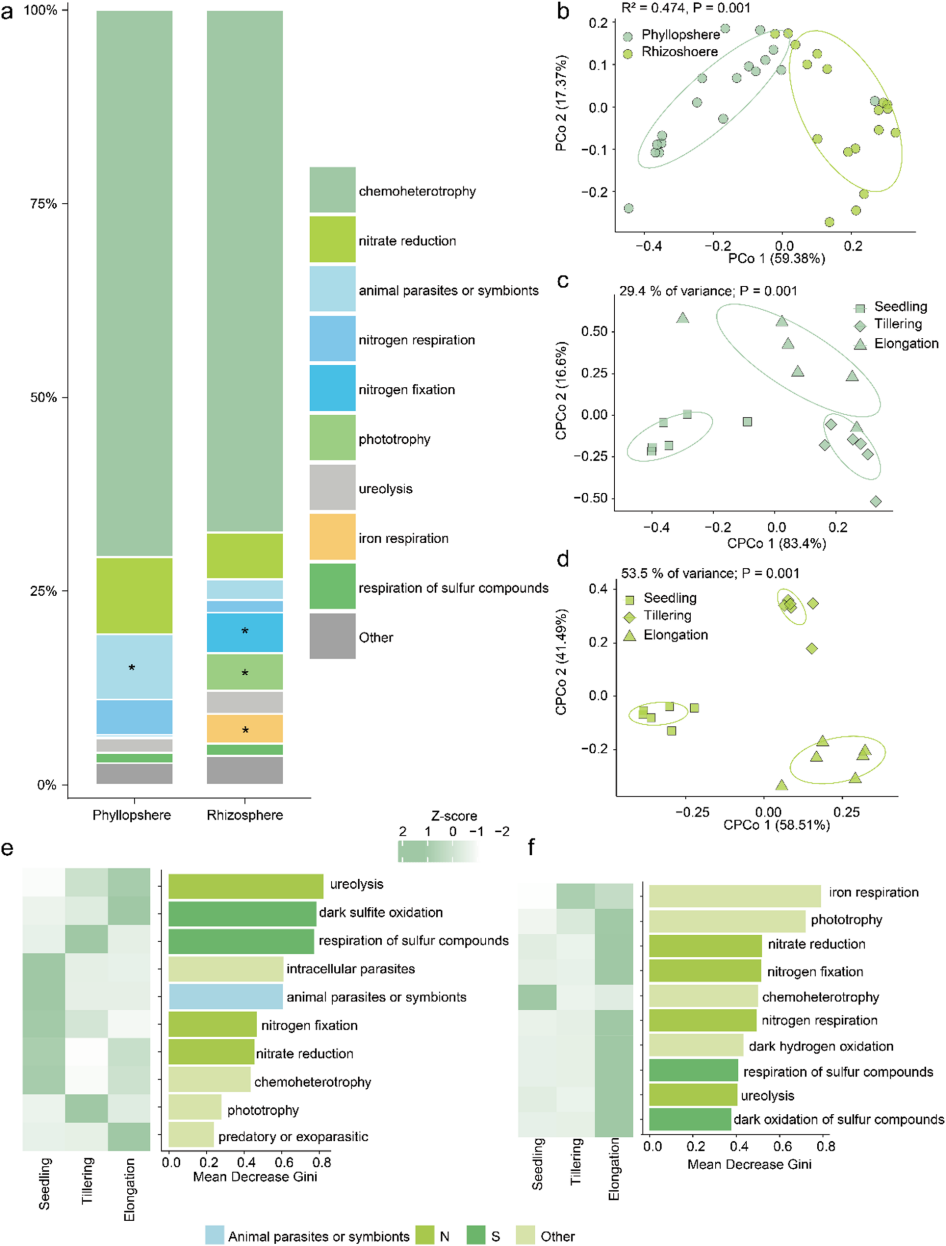
Predictive functional analysis of the phyllosphere and rhizosphere microbiome of rice. **(a)** Functional distribution of bacteria in the rhizosphere and phyllosphere microbiomes. Asterisks represent significant differences between the phyllosphere and rhizosphere as assessed with an unpaired two sides t-test (*p* < 0.05). **(b)** Unconstrained PCoA (for principal coordinates PCo1 and PCo2) based on Bray-Curtis distances showing functions of bacterial community clustering for the phyllosphere and rhizosphere (p value was calculated by one-way PERMANOVA). Constrained PCoA based on Bray-Curtis distances showing separation of functional profiles for seedling, tillering, and elongation stages in (**c**) the phyllosphere and (**d**) the rhizosphere, respectively (p value was calculated with PERMANOVA). Identified functions that can distinguish between developmental stages for (**e**) the phyllosphere and (**f**) the rhizosphere. Identified features are based on Mean Decrease Gini of random forest models. The heatmap represent their enrichment at three developmental stages

PCOA analysis indicated that the predicted functional composition significantly differed between the phyllosphere and rhizosphere microbiome (Fig. 3b; *p* = 0.001, R^2^ = 0.474). Further PERMANOVA analysis and CPCOA ordinations indicated that developmental stages explained 29.4% of the variation for the functional composition in the phyllosphere microbiome (Fig. 3c). Whereas 53.5% of the variation in the functional composition of the rhizosphere microbiome was explained by developmental stages (Fig. 3d). These findings indicated that the functions of the rice microbiome were influenced by compartments and developmental stages. The predominant bacterial functions in both phyllosphere and rhizosphere were comprised by chemoheterotrophy (70.6% and 67.5% of relative abundance for phyllosphere and rhizosphere, respectively), followed by nitrate reduction (10% and 6%), APS (potential animal parasites or symbionts, 8.4% and 2.7%), nitrogen respiration (4.6% and 1.6%), nitrogen fixation (0.3% and 5.2%), phototrophy (0.1% and 4.9%), ureolysis (1.9% and 3%), iron respiration (0.03% and 5%) and respiration of sulfur compounds (1.3% and 1.6%) (Fig. 3a). Compared to the phyllosphere, the rhizosphere had a higher relative abundance of nitrogen fixation, iron respiration and phototrophy, while the phyllosphere had a higher relative abundance of APS (Fig. 3a, Supplementary Table 5; *p* < 0.05).

Random Forest supervised learning models were further employed to identify which functional composition can explain the variations during rice developmental stages. Specifically, functions belonging to APS, N-cycle (ureolysis, nitrogen fixation, nitrate reduction), S-cycle (dark sulfite oxidation and respiration of sulfur compounds), intracellular parasites, predators or exoparasites, chemoheterotrophy, and phototrophy were the top features in the classification models for developmental stages in the rice phyllosphere (Fig. 3e). Moreover, APS, intracellular parasites, nitrogen fixation, nitrate reduction, and chemoheterotrophy were enriched during the seedling stage (Fig. 3e). Functions related to respiration of sulfur compounds and phototrophy were enriched in the tillering stage (Fig. 3e). Ureolysis, dark sulfite oxidation, and predators or exoparasites were enriched in the elongation stage (Fig. 3e). When classifying the functions for developmental stages in the rice rhizosphere, N-cycle (nitrate reduction, nitrogen fixation, nitrogen respiration and ureolysis), S-cycle (respiration of sulfur compounds and dark oxidation of sulfur compounds), iron respiration, phototrophy, chemoheterotrophy, and dark hydrogen oxidation were top features (Fig. 3f). Chemoheterotrophy and iron respiration were enriched in the seedling and tillering stage, respectively. Other functions from the top features were enriched in the elongation stage (Fig. 3f). Taken together, these results confirmed that plant developmental stages not only differentially influenced the microbial taxonomy but also the functional repertoires in the phyllosphere and rhizosphere of rice grown in soil from the urban area.

### The effect of developmental stages and urban area on the presence of potential animal parasites or symbionts in the rice Microbiome

Further analysis revealed that bacteria associated with the human gut and human pathogens were the primary contributors to microbes typically classified as APS (potential animal parasites or symbionts) in the phyllosphere and rhizosphere of rice cultivated in soil from the urban area (Fig. S6). In addition, the taxonomy of APS inhabiting the phyllosphere significantly differed from those inhabiting the rhizosphere (Fig. S7; R^2^ = 0.343, *p* = 0.001). PERMANOVA analysis and CPCoA ordinations indicated that plant developmental stages explained 21.7% of the variation in terms of the taxonomy of APS in the phyllosphere (Fig. S7). In contrast, 40% of the same variation was explained by plant developmental stage in the rhizosphere. (Fig. S7). These findings indicate that the composition of APS can be influenced by plant developmental stages.

**Fig. 4:**
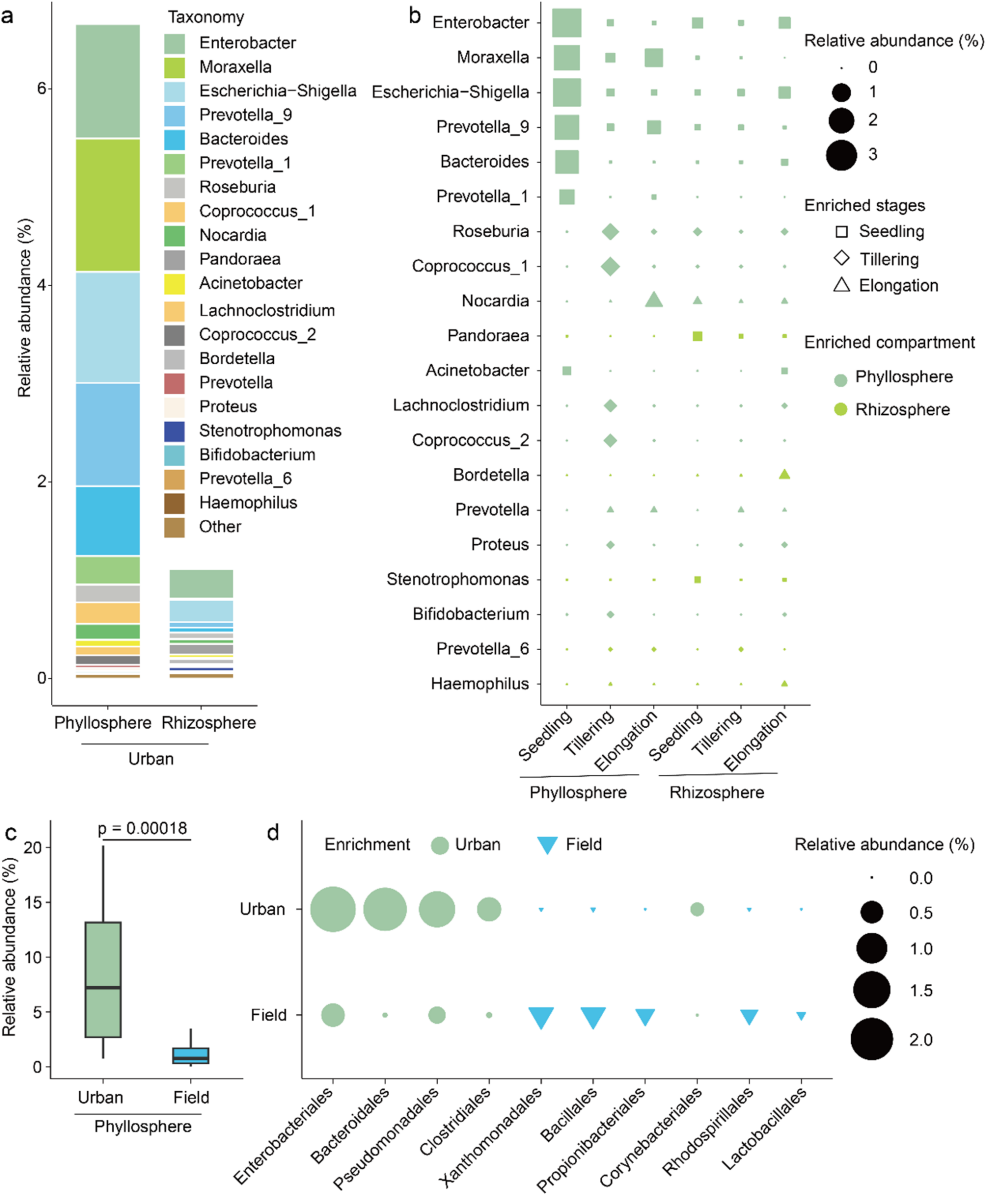
Bacteria assigned to potential animal parasites or symbionts in the phyllosphere and rhizosphere of rice grown in urban area soil and in the field. (**a**) Genus-level distribution of APS (potential animal parasites or symbionts) in the rhizosphere and phyllosphere of rice grown in urban area soil. (**b**) The dynamics of APS at seedling, tillering and elongation stages in the rhizosphere and phyllosphere. Relative abundance (**c**) of and order-level distribution (**b**) of APS in the phyllosphere of rice grown either in urban area soil or in the field

Furthermore, taxonomic analysis of APS showed that the predominant bacterial genera in both the phyllosphere and rhizosphere were comprised of *Enterobacter* (1.17% and 0.30% of relative abundance for phyllosphere and rhizosphere, respectively), followed by *Moraxella* (1.36% and 0.01%), *Escherichia-Shigella* (1.13% and 0.22%), *Prevotella* 9 (1.05% and 0.06%), *Bacteroides* (0.71% and 0.05%), *Prevotella* 1 (0.29% and 0.0002%), *Roseburia* (0.18% and 0.06%), *Coprococcus* 1(0.22% and 0.01%), and *Nocardia* (0.16% and 0.05%) (Fig. 4a). Further analysis showed that the predominant bacterial genera within this group, including *Enterobacter*, *Moraxella*, *Escherichia-Shigella*, *Prevotella* 1, *Prevotella* 9, *Bacteroides*, and *Acinetobacter* were enriched at the seedling stage in the phyllosphere (Fig. 4b). Remarkably, the phyllosphere also enriched bacterial genera such as *Roseburia*, *Coprococcus* 1, *Coprococcus* 2, *Lachnoclostridium*, *Proteus*, and *Bifidobacterium* at the tillering stage (Fig. 4b). However, we found that genera such as *Pandoraea*, *Stenotrophomonas*, *Prevotella* 6, *Bordetella*, and *Haemophilus* were enriched in the rhizosphere (Fig. 4b). Analogous trends were also shown at the bacterial order level (Fig. S8).

Based on these results, we found that the rice phyllosphere from plant grown in urban areas may enrich bacteria classified as APS. To further compare the impact of the urban area on plant microbiome assembly, we compared the phyllosphere microbiome of rice (variety: ZH11) grown in soil of an urban area with that of rice (7 varieties: Chakhao, Phouren-mubi, Phoungang, Tolenphou, Moirangphou, Moirangphou khokngangbi, and High Yielding variety) grown in the field [38]. We found that the microbial composition in the field was dominated by Rhizobiales (27%), Enterobacteriales (26%), Sphingomonadales (18.6%), Pseudomonadales (6.9%), Cytophagales (4.1%), Micrococcales (3.2%), and Burkholderiales (2.4%) (Fig. S9). Community structure comparisons indicated significant differences in the composition of phyllosphere microbial communities between rice grown in soil of the urban area and in the field (Fig. S9). Differential analysis revealed that the relative abundance of Rhizobiales, Enterobacteriales, Sphingomonadales, Cytophagales, and Micrococcales was significantly higher in the phyllosphere of rice grown in the field than in that grown in urban area soil, while the relative abundance of Bacteroidales, Clostridiales, and Burkholderiales was significantly higher in the phyllosphere of rice grown in urban area soil (Fig. S9). The functional composition of the phyllosphere microbiome of rice grown in the field mainly consisted of chemoheterotrophy (75.4%), ureolysis (19.7%), APS (1.1%), plant pathogens (0.87%), and nitrate reduction (0.76%) (Fig. S10). Additionally, we found that the relative abundance of ureolysis and plant pathogens was significantly higher in the phyllosphere of rice grown in the field than in that grown in urban area soil, while the relative abundance of APS and nitrate reduction was significantly higher in the phyllosphere of rice grown in soil of the urban area (Fig. S10). These results indicated that urban area soil influences the composition and function of the rice phyllosphere microbial community and provided additional confirmation that the enrichment of APS in the phyllosphere is caused by growth in the urban area soil.

Based on the significant difference in the occurrence of bacteria assigned to APS in the phyllosphere between rice grown in urban area and field (Fig. 4D), we further analyzed their taxonomic composition. At the order level, APS belonging to Enterobacteriales, Bacteroidales, Pseudomonadales, and Clostridiales were predominantly enriched in the phyllosphere of rice grown in urban area soil. Conversely, APS belonging to Xanthomonadales, Bacillales, Propionibacteriales, Rhodospirillales, and Lactobacillales were enriched in the phyllosphere of rice grown in field soil (Fig. 4D). At the genus level, APS belonging to *Moraxella*, *Enterobacter*, *Escherichia-Shigella*, *Prevotella*, *Bacteroides*, *Roseburia*, *Coprococcus*, *Nocardia*, *Proteus*, and *Lachnoclostridium* were enriched in the phyllosphere of rice grown in urban area soil, while *Pantoea*, *Staphylococcus*, *Pseudomonas*, *Roseomonas*, *Stenotrophomonas*, *Propionibacterium* and *Streptococcus* were enriched in the phyllosphere of rice grown in field soil (Fig. S11). These results indicate that the enrichment of APS in the phyllosphere of rice grown in urban area soil is primarily due to Enterobacteriales, Bacteroidales, Pseudomonadales, and Clostridiales. To further determine the effects of environmental factors, plant genetics, and plant species on the enrichment of APS (potential animal parasites or symbionts) in plant phyllosphere and rhizosphere, we compared the phyllosphere microbiome of camphor trees grown in urban areas [39], the rhizosphere [40] and phyllosphere [13]microbiomes of ZH11 grown in urban/field conditions, and the phyllosphere microbiome of seven different rice varieties [38] grown in the field. The results showed that APS in the rhizosphere and phyllosphere microbiomes of urban are soil-grown samples were significantly higher than those in field-grown samples (Fig. S12). Overall, we found that both urban area and developmental stages have significant impacts on the composition of bacterial populations assigned to APS.

### The effects of potential animal parasites or symbionts and development stages on the rice Microbiome assembly of phyllosphere and rhizosphere in an urban area

Random Forest supervised learning models revealed that the top features in the classification models for developmental stages of phyllosphere and rhizosphere microbiome of rice grown in soil of an urban area were showed substantial alterations, when in silico depletion of APS (potential animal parasites or symbionts) (Fig. S13, Fig. 2c and d). Only OTU168 and OTU719 belonging to the order Sphingobacteriales and enriched during the elongation stage were identified as the top feature both in APS-free and APS-containing rhizosphere datasets (Fig. S13). We further performed co-occurrence network analysis to assess the impact of microbiome interaction after in silico depletion of APS. Our results showed that nodes and connections (average degree) of microbial networks in APS-free microbiomes and APS-containing microbiomes showed low differences at the three developmental stages in the rhizosphere (Fig. S14). However, when in silico depletion of APS, the clustering coefficient of the microbial network was substantially alternated at the seedling and elongation stages in the phyllosphere (Fig. S14). We further compared the “network hubs” between the microbial networks obtained with APS-free and APS-containing microbiomes. Without APS, the hubs were similar for the three developmental stages in the rhizosphere. Notably, one new hub (belonging to the bacterial order Pseudomonadales) and one new hub (assigned to Bacteroidales) were identified at the seedling and tillering stages in the APS-free microbiome network of the phyllosphere, respectively (Fig. S14). Taken together, these results confirmed that the APS differentially affects the composition and interaction of the microbiome in the phyllosphere and rhizosphere. Therefore, we hypothesize that, similar to developmental stages, bacteria that are commonly assigned to APS may also contribute to microbiome assembly.

**Fig. 5:**
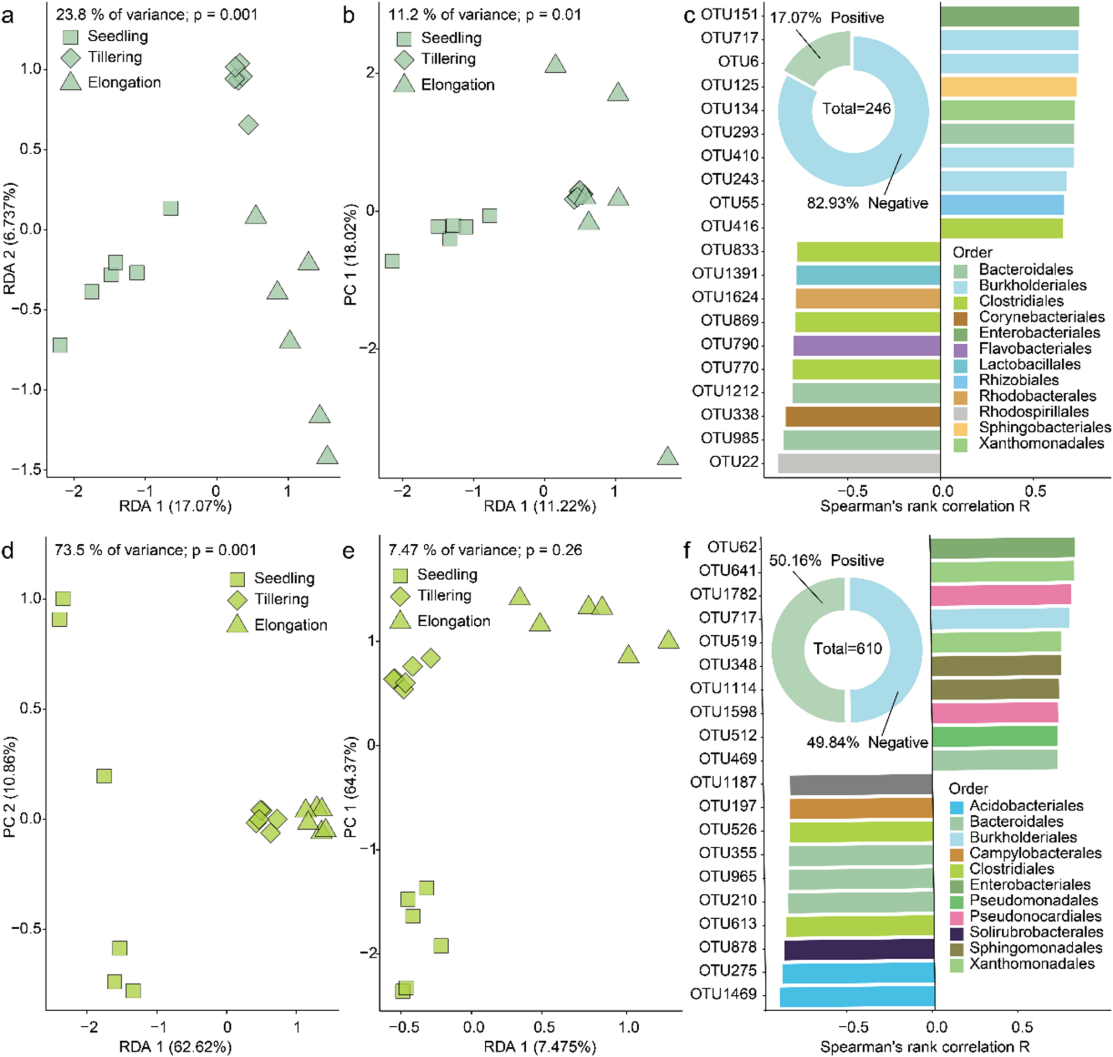
Comparative assessments of the effects of developmental stages and potential animal parasites or symbionts on the phyllosphere and rhizosphere microbiome. RDA (Redundancy Analysis) was used to link the APS (potential animal parasites or symbionts)-free phyllosphere microbiome to (**a**) developmental stages and (**b**) APS, respectively (p-value was calculated through one-way PERMANOVA). RDA was used to link the APS-free rhizosphere microbiome to (**d**) developmental stages and (**e**) APS, respectively (p-value was calculated through one-way PERMANOVA). The correlation analysis between APS and APS-free microbiome in the rice (**c**) phyllosphere and (**f**) rhizosphere. The pie chart indicates the total number of OTUs that are significantly correlated with APS in the APS-free microbiome. The bar plot shows the top 10 APS-responsive OTUs with significant positive and negative correlations. Spearman’s rank correlation R values were calculated through two-sided Spearman coefficient

We conducted redundancy analysis (RDA) to compare the effects of developmental stages and APS on the microbiome of rice grown in soil of an urban area. We found that the developmental stages explained 23.8% (Fig. 5a; *p* = 0.001) and 73.5% (Fig. 5d; *p* = 0.001) of the total microbiome variation in the phyllosphere and rhizosphere, respectively. Moreover, APS explained 11.2% (Fig. 5b; *p* = 0.01) and 7.47% (Fig. 5e; *p* = 0.26) of the total microbiome variation in the phyllosphere and rhizosphere, respectively. This observation suggests that the developmental stages of rice have a more pronounced impact on the assembly of the microbiome than APS.

Additionally, correlation analysis revealed that 42 OTUs (17.07% of the total) were significantly positively correlated with APS in the phyllosphere of rice grown in urban area soil, whereas 204 OTUs (82.93% of the total) exhibited a significant negative correlation with them (Fig. 5c, Supplementary Data 2; *p* < 0.05). The most significant positive and negative correlations were OTU151 (*R* = 0.74; belonging to the order Enterobacteriales) and OTU22 (*R* = -0.87; Rhodospirillales), respectively (Fig. 5c, Supplementary Data 2). However, in the rhizosphere of rice grown in urban area soil, 306 OTUs (50.16%) correlated positively and 304 OTUs (49.84%) negatively with APS (Fig. 5f, Supplementary Data 2; *p* < 0.05). The top OTUs exhibiting significant positive and negative correlations were OTU62 (*R* = 0.85; Enterobacteriales ) and OTU1469 (*R* = -0.90; Acidobacteriales), respectively. (Fig. 5f, Supplementary Data 2). These results demonstrated that bacteria assigned to APS have different effects on the microbiome in the rice phyllosphere and rhizosphere. In conclusion, APS had a stronger effect on microbiome variation in the phyllosphere and showed more correlations with other microbiome members in the rhizosphere.

## Discussion

Plant compartments are considered as one of the main host factors influencing microbiome assembly [30, 33]. This was confirmed by the present study. Our results demonstrated that diversity of the rhizosphere microbiome was significantly higher than that in the phyllosphere. These findings are consistent with previous studies conducted with rice [57], maize [33], strawberry [36], and various cereal plants [58] showing that belowground microbiome is more diverse than the aboveground one.

We further found that developmental stages significantly affected both the rhizosphere and phyllosphere microbiomes, with a greater contribution to the variation of the rhizosphere microbiome compared to the phyllosphere. Of note, many previous studies showed that the developmental stage can explain a more substantial proportion of the variation within belowground compartments than in aboveground compartments of soybean [59], grapevine [34], and Arabidopsis thaliana [60]. Additionally, we showed that developmental stages differentially impact microbial interaction networks in the phyllosphere and rhizosphere. For instance, the connectivity of phyllosphere microbial interactions increased during the tillering stage and decreases during the elongation stage, whereas the network connectivity of rhizosphere microorganisms gradually increased with developmental stages, reaching a highly complex state during the elongation stage. The observed, dynamic pattern of microbial interaction networks in the rhizosphere is largely consistent with a field study conducted with rice [61]. However, bacterial connectivity in the phyllosphere microbiome gradually decreased with developmental stages in field growth maize [33]. Due to the cultivation of rice in a glasshouse in this study, the sources of phyllosphere microbiota (such as animal vectors and rainwater) were reduced. Therefore, phyllosphere was mainly constituted by microbes from the soil.

Furthermore, developmental stages significantly and differently affected the functional profiles in the rhizosphere and phyllosphere microbiome. For example, functions related to APS (potential animal parasites or symbionts) and the N-cycle peaked during the seedling stage in the phyllosphere and then decreased with developmental stages, while functions related to the N-cycle and S-cycle showed a tendency to accumulate in the rhizosphere with developmental stages, reaching their peak during the elongation stage. Similarly, previous field experiments have suggested that rice can actively recruit bacteria to modulate the nitrogen cycle in the rhizosphere [32, 40]. The abundance and composition of the plant exudates and metabolites and immune response can influence microbial patterns within the rhizosphere and phyllosphere [15, 62–65]. The host can more readily recruit microbes and related functions from the soil in rhizosphere, which may be one of the reasons for the differential assembly of phyllosphere and rhizosphere microbiota at different developmental stages.

Previous research has indicated that the phyllosphere microbiome assembly is more strongly influenced by anthropogenic activities than the soil microbiome [26, 27, 66]. In the present study, the relative abundance of APS in the phyllosphere of rice grown in urban area soil was significantly higher than that in the rhizosphere. Additionally, bacteria assigned to APS in the phyllosphere were also shown to be influenced by the developmental stage. Their relative abundances were highest during the seedling stage and decreased during the tillering and elongation stages in the phyllosphere. *Enterobacter*, *Escherichia-Shigella*, *Prevotella* and *Bacteroides* are widely found in the human gut [67, 68]. *Moraxella* is found in the nasal microbiota of urban children [69] and in the indoor air [70]. This indicates that bacteria assigned to APS in rhizosphere and phyllosphere of rice grown in soil from the urban area may have been enriched by human activities. Soil and phyllosphere microbiomes were previously shown to be influenced by plant species in urban greenspaces [24, 71]. This indicates that APS in the rhizosphere and phyllosphere of rice grown in urban areas may be selected by the host, which may cause an increase or decrease in the abundance of these microorganisms as the plant grows.

Compared to rice grown in fields, we found that the urban area can have a significant impact on the assembly of the rice phyllosphere microbiome. Specifically, the phyllosphere microbiome of rice grown in urban area soil enriched the bacterial order Bacteroidales and the functions related to APS. In contrast, the bacterial order Rhizobiales and the nitrogen cycling function ureolysis were enriched in rice grown in field soil. Bacteroidota and Bacillota were identified as the dominant bacterial phyla shared across the microbiomes of soils, plants, animals, and humans [16]. It is currently assumed that soil serves as a source of microbiomes for both animals and plants [16]. APS might be transferred between animals and plants via soil. This variation in the phyllosphere microbiome between field and urban environments may be influenced by distinct anthropogenic activities. The use of chemical fertilizers, intensive fertilization, and agronomic management plays an important role in shaping the phyllosphere assembly in field-grown rice [72, 73]. Different irrigation water sources also substantially impact the microbiome in both field and urban environments [74, 75], which may be the reason for the enrichment of APS in urban-grown rice.

We also found that APS influence the assembly of microbial communities in the phyllosphere of rice grown in urban area soil. When in silico depletion of APS, the effect on the phyllosphere was greater than that on the rhizosphere. Notably, the phyllosphere microbial network structure and hubs were changed in the seedling stage. Further investigation into the impact of APS on microbiome variation revealed that APS are mainly negatively correlated with phyllosphere microbes. These results suggested that some APS may be antagonized by other plant microbiome members at different developmental stages, while some APS may have a better ability to colonize specific compartments of plants. The potential of the plant microbiota to antagonize human pathogens was previously demonstrated in a study with two model indoor plants [76], providing further indication that our data reflects general trends of APS dynamics in the plant microbiome. Future studies with a more comprehensive classification of animal parasites or symbionts are needed to improve the accuracy of their effects on soil, plant, animal, and human microbiomes. More research on the interactions between animal-plant symbiotic microbes and plant- and animal-microbiomes is crucial for establishing a better understanding of microbiome interconnectivity in ‘*One Health*’.

## Conclusion

Our study provides new insights into microbiome assembly during different development stages in plants grown in urban area soil. Plant developmental stages differentially influenced the community and functional compositions of the phyllosphere and rhizosphere microbiome of rice grown in urban area soil. Moreover, our data showed that potential animal parasites or symbionts are major microbiome components influenced by urban area soil. One of the most pronounced features was the enrichment of Bacteroidales in the phyllosphere. Furthermore, potential animal parasites or symbionts were shown to significantly influence the assembly of the phyllosphere microbiome.

## Electronic supplementary material

Below is the link to the electronic supplementary material.

Supplementary Material 1

Supplementary Material 2

Supplementary Material 3

## Data Availability

Raw sequence data (16S rRNA gene fragment sequencing) generated in this study have been deposited in the Genome Sequence Archive of China National Center for Bioinformation (CNCB) [77] under accession PRJCA033722. Plant microbiome datasets obtained from urban and field samples were obtained from NCBI (BioProject: PRJNA1158963 [38] and PRJNA919006 [39]), CNCB (PRJCA016320 [13] and PRJCA001214 [40]). Scripts employed in the computational analyses are available on Github [https://github.com/PengQianze/].

## Abbreviations

PCoA: Principal coordinates analysis
CPCoA: Constrained principal coordinates analysis
OTU: Operational taxonomic unit
APS: Potential animal parasites or symbionts
PERMANOVA: Permutational multivariate analysis of variance
MS: Murashige and Skoog
NaOCl: Sodium hypochlorite
EtOH: Ethanol
HHRRC: Hunan Hybrid Rice Research Center
PBS: Phosphate buffer solution
RDA: Redundancy analysis
FAPROTAX: Functional annotation of prokaryotic taxa
