# Supplementary material for: The effect of developmental stages on microbiome assembly in the phyllosphere and rhizosphere of rice grown in urban area soil
Source: Environ Microbiome. 2025 Jul 11;20:86. doi: 10.1186/s40793-025-00748-9 (PMC12255072; doi:10.1186/s40793-025-00748-9)
Supplement: Supplementary file 3 — Supplementary Material 3 [file 40793_2025_748_MOESM3_ESM.docx]

**The effect of developmental stages on microbiome assembly in the phyllosphere and rhizosphere of rice grown in urban area soil**

Peng *et al.*

**Supplementary Table 1. Information related to microbiome datasets used in this study.**

| BioProject ID | Host | Variety | Compartment | Location | Source of samples |
| --- | --- | --- | --- | --- | --- |
| PRJNA919006 | camphor trees | - | phyllosphere | Ningbo, China (29.9997 N, 121.8392 E) | Urban |
| PRJCA033722 | rice | ZH11 | phyllosphere | Changsha, China (28.1958 N, 113.0838 E) | Urban |
| PRJCA033722 | rice | ZH11 | rhizosphere | Changsha, China (28.1958 N, 113.0838 E) | Urban |
| PRJCA016320 | rice | ZH11 | phyllosphere | Taojiang, China (28.6358 N, 112.0158 E) | Field |
| PRJNA1158963 | rice | Chakhao, Phouren-mubi, Phoungang, Tolenphou, Moirangphou, Moirangphou Khokngangbi, and High Yielding variety | phyllosphere | Manipur, India (24.6637 N, 93.9063 E) | Field |
| PRJCA001214 | rice | ZH11 | rhizosphere | Changping, China (40.109 N, 116.424 E) | Field |

**Supplementary Table 2. Assessment of differences in relative abundance of prevalent bacterial orders between the phyllosphere and rhizosphere.**

| Order | Phyllosphere | Rhizospere | p value |
| --- | --- | --- | --- |
| Bacteroidales | 32.916±5.236 | 1.023±0.118 | 0.000012 |
| Clostridiales | 19.322±3.216 | 2.468±0.757 | 0.0000646 |
| Burkholderiales | 9.584±2.547 | 11.241±1.647 | 0.589 |
| Rhizobiales | 2.657±0.877 | 15.235±0.773 | 2.08E-12 |
| Acidobacteriales | 0.168±0.091 | 16.719±1.186 | 8.61E-11 |
| Xanthomonadales | 0.169±0.073 | 12.705±1.543 | 0.00000029 |
| Rhodospirillales | 1.42±0.738 | 7.632±0.75 | 0.00000115 |
| Sphingobacteriales | 0.666±0.275 | 7.094±0.466 | 2.32E-12 |
| Pseudomonadales | 5.024±2.477 | 0.285±0.09 | 0.0729 |

Note: p values were calculated with an unpaired two sides t-test. Values are means ± SE of relative abundance (%). The numbers of replicated samples are as follows: Phyllosphere (n=18) and Rhizosphere (n=18).

**Supplementary Table 3. Assessment of differences in alpha diversity in the phyllosphere between three development stages.**

| Alpha diveristy | Seedling | Tillering | Elongation |
| --- | --- | --- | --- |
| richness | 21.5±2.884 b | 57.667±1.944 a | 16.167±2.33 b |
| chao1 | 24.508±4.369 b | 126.567±23.65 a | 27.417±5.219 b |
| shannon | 2.624±0.143 b | 3.825±0.037 a | 2.117±0.19 b |
| simpson | 0.895±0.016 b | 0.971±0.002 a | 0.825±0.028 b |

Note: Different letters indicate a significant difference according to an unpaired two sides t-test. Values are means ± SE of relative abundance (%). The numbers of replicated samples are as follows: Phyllosphere (n=18) and Rhizosphere (n=18).

**Supplementary Table 4. Assessment of differences in alpha diversity in rhizosphere between three development stages.**

| Alpha diveristy | Seedling | Tillering | Elongation |
| --- | --- | --- | --- |
| richness | 40±1.125 b | 62.333±1.926 a | 65.5±3.019 a |
| chao1 | 79.776±10.046 b | 129.682±11.58 a | 163.818±13.975 a |
| shannon | 3.206±0.063 b | 3.964±0.048 a | 3.953±0.079 a |
| simpson | 0.932±0.008 b | 0.977±0.002 a | 0.972±0.004 a |

Note: Different letters indicate a significant difference according to unpaired two sides t-test. Values are means ± SE of relative abundance (%). The numbers of replicated samples are as follows: Phyllosphere (n=18) and Rhizosphere (n=18).

**Supplementary Table 5. Assessment of differences in the relative abundance of functions in the phyllosphere and rhizosphere microbiome.**

| Functions | Phyllosphere (%) | Rhizosphere (%) | p value |
| --- | --- | --- | --- |
| chemoheterotrophy | 70.61±3.34 | 67.45±4.12 | 0.556 |
| nitrate reduction | 9.97±1.72 | 6.03±1.37 | 0.0825 |
| animal parasites or symbionts | 8.37±1.52 | 2.66±0.33 | 0.00169 |
| nitrogen respiration | 4.6±1.61 | 1.62±0.39 | 0.0887 |
| predatory or exoparasitic | 2.06±1.38 | 0.73±0.1 | 0.35 |
| ureolysis | 1.87±0.52 | 2.98±0.65 | 0.191 |
| respiration of sulfur compounds | 1.34±0.9 | 1.59±0.53 | 0.812 |
| nitrogen fixation | 0.3±0.1 | 5.22±0.48 | 7.44E-09 |
| plant pathogen | 0.24±0.11 | 0.59±0.1 | 0.023 |
| dark oxidation of sulfur compounds | 0.19±0.09 | 0.72±0.22 | 0.0365 |
| nitrification | 0.12±0.07 | 0.28±0.06 | 0.081 |
| phototrophy | 0.1±0.07 | 4.85±1.22 | 0.0012 |
| dark iron oxidation | 0.1±0.07 | 0.14±0.06 | 0.691 |
| intracellular parasites | 0.06±0.05 | 1.24±0.21 | 3.63E-05 |
| iron respiration | 0.03±0.03 | 3.84±1.17 | 0.00457 |
| dark sulfite oxidation | 0.02±0.01 | 0±0 | 0.0217 |
| dark hydrogen oxidation | 0.01±0 | 0.03±0.01 | 0.075 |

Note: p values were calculated with an unpaired two sides t-test. Values are means ± SE of relative abundance (%). The numbers of replicated samples are as follows: Phyllosphere (n=18) and Rhizosphere (n=18).


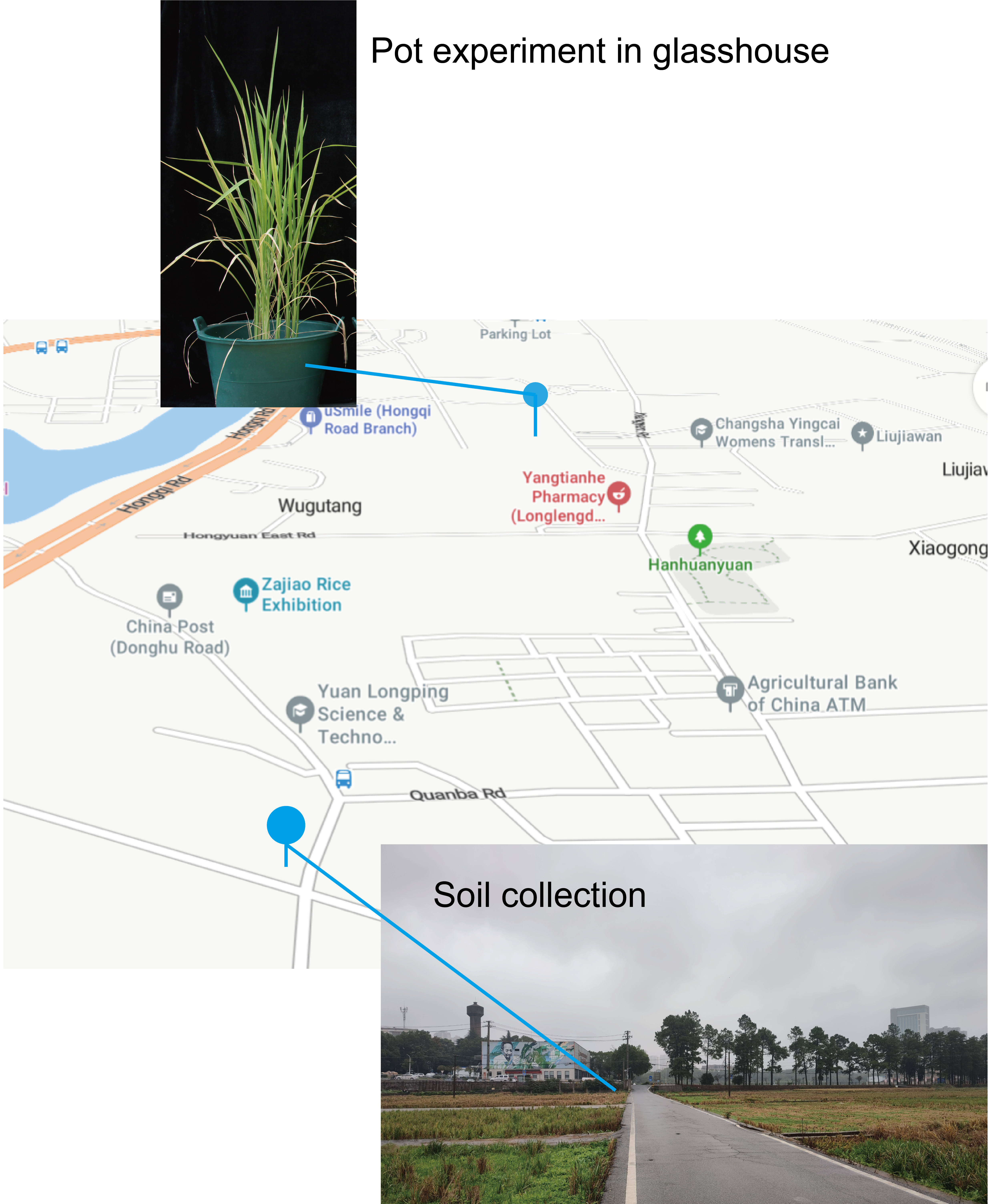


**Supplementary Fig. 1.** Sites of soil collection (28°11′44.963″ N, 113°05′1.684″ E) and pot experiments (28°12′9.4″ N, 113°05′10.72″ E).


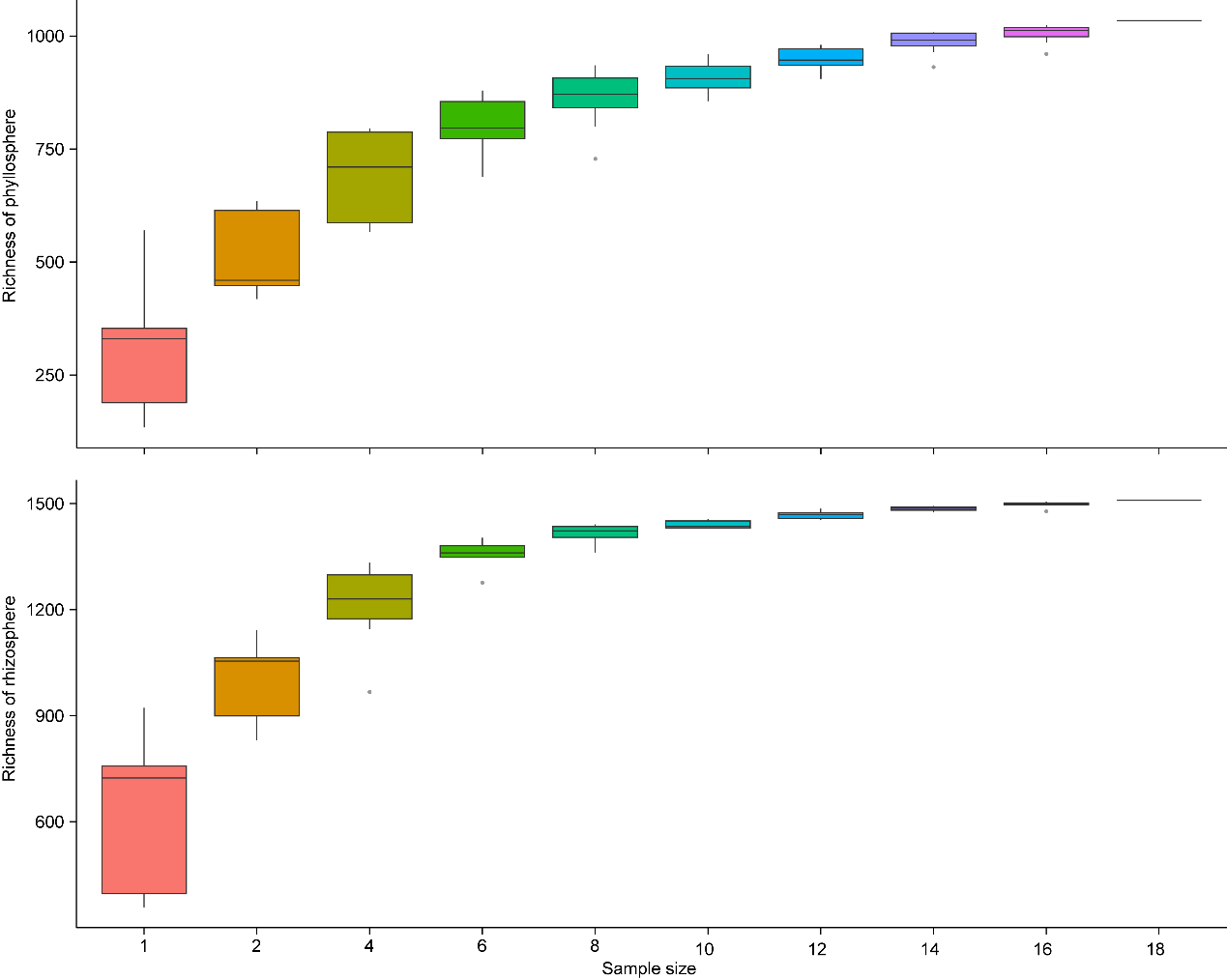


**Supplementary Fig. 2.** Rarefaction curves of richness (observed OTUs) reach the saturation stage with increasing numbers of samples. The tops and bottoms of boxes represent 75th and 25th percentiles, respectively. The upper and lower whiskers extend to data no more than 1.5 the interquartile range from the upper edge and lower edge of the box, respectively. The numbers of samples are as follows: phyllosphere (n=18) and rhizosphere (n=18).


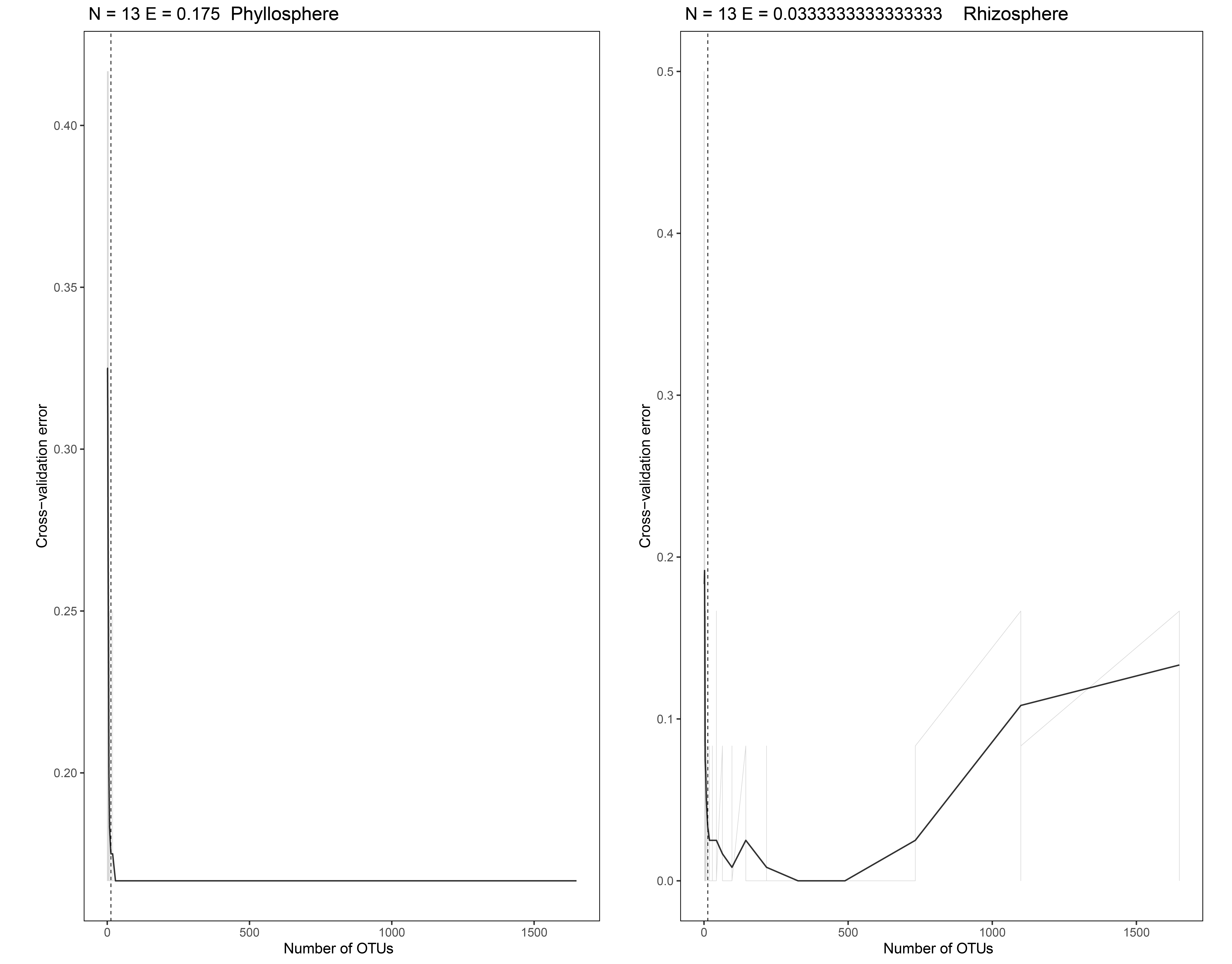


**Supplementary Fig. 3.** Accuracy of random forest algorithms between the phyllosphere and rhizosphere microbiome of rice grown in urban area soil. The optimal number of OTUs and the cross-validation error of the RF model for the phyllosphere microbiome were 13 and 0.175, respectively. The optimal number of OTUs and the cross-validation error of the RF model for the rhizosphere microbiome were 13 and 0.033, respectively. Optimal number of OTUs were calculated by ‘randomForest’ package.





**Supplementary Fig. 4.** Network nodes assigned to the indicated bacterial orders at seedling (**a**), tillering (**b**) and elongation (**c**) stages in the phyllosphere microbiome of rice grown in urban area soil.

**
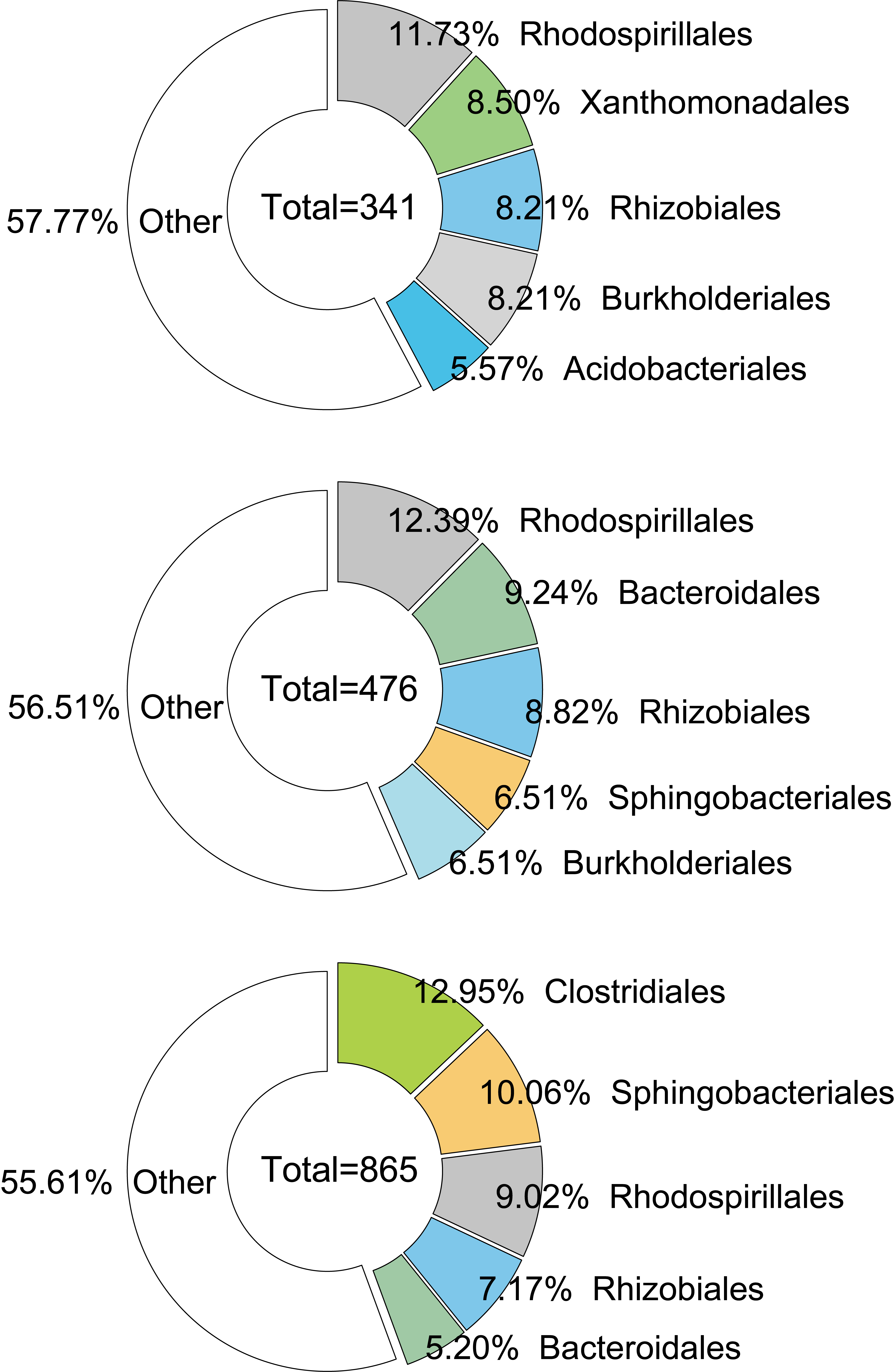
**

**Supplementary Fig. 5.** Network nodes belonging to the indicated bacterial orders at seeding (**a**), tillering (**b**) and elongation (**c**) stages in the rhizosphere microbiome of rice grown in urban area soil.


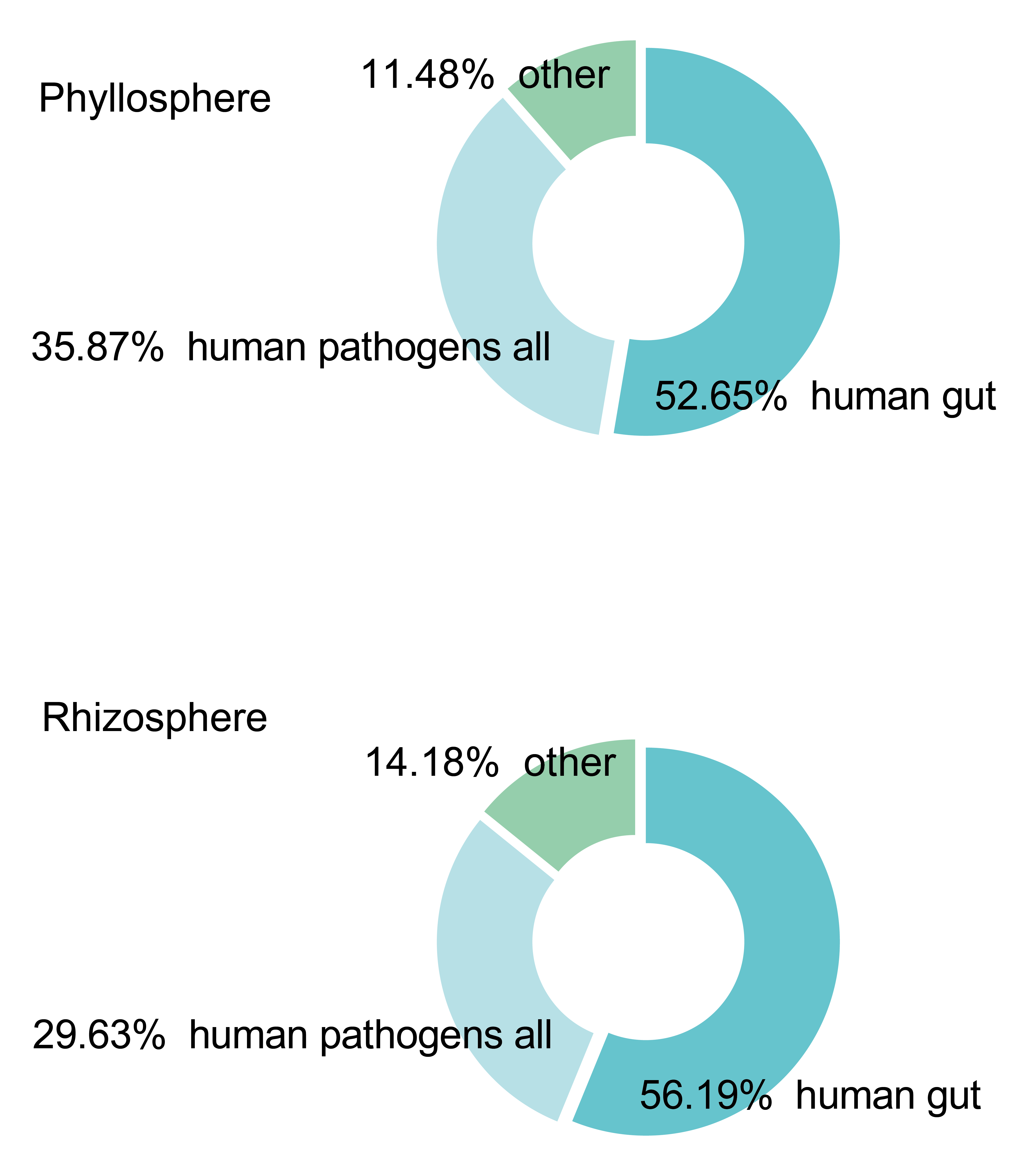


**Supplementary Fig. 6.** The distribution of potential animal parasites or symbionts in the phyllosphere and rhizosphere of rice grown in urban area soil.


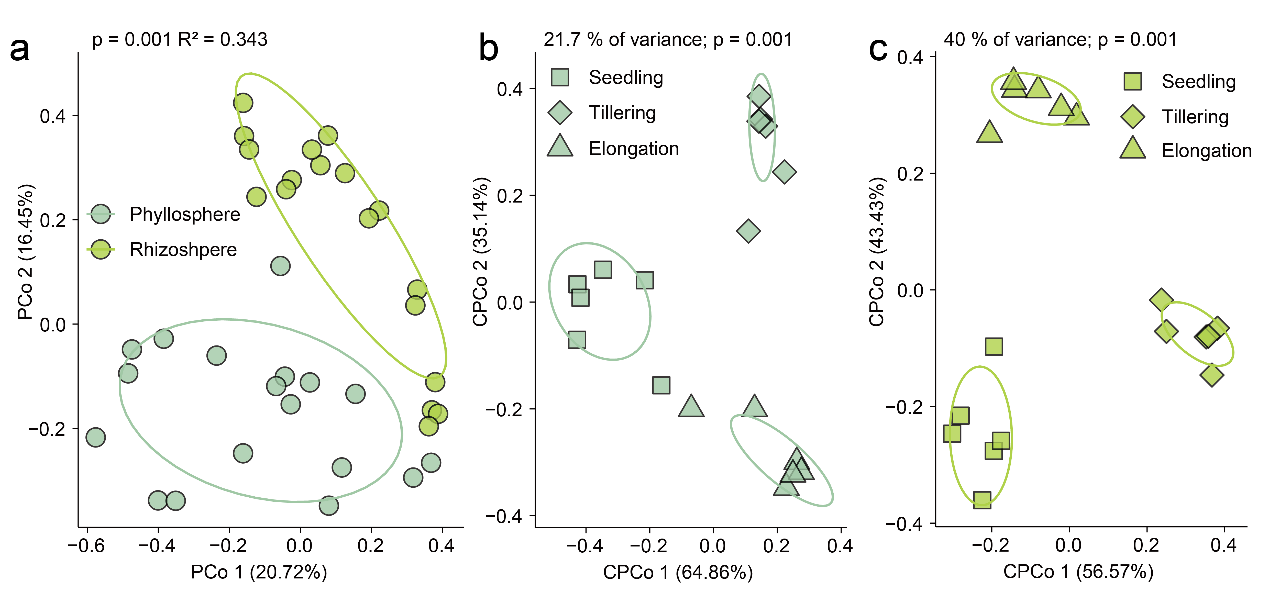


**Supplementary Fig. 7.** PCoA and CPCoA of APS (potential animal parasites or symbionts) of rice grown in urban area soil. (**a**) Unconstrained PCoA (for principal coordinates PCo1 and PCo2) based on Bray-Curtis distances showing APS clustering in the phyllosphere and rhizosphere (P-value was calculated by one-way PERMANOVA). Constrained PCoA based on Bray-Curtis distances showing APS separation for seeding, tillering and elongation development stages at (**b**) Phyllosphere and (**c**) rhizosphere, respectively (p-value was calculated with PERMANOVA).


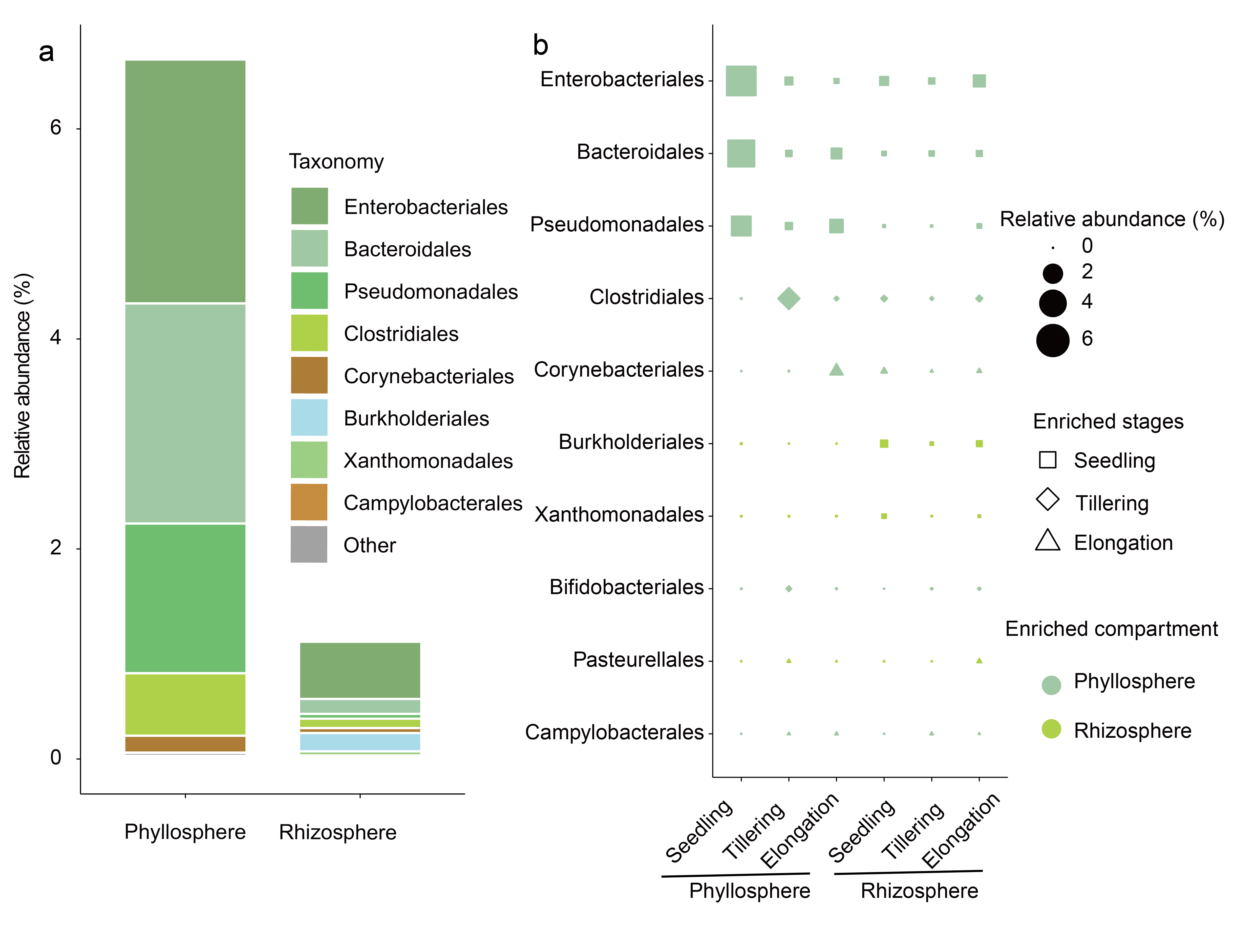


**Supplementary Fig.** **8.** Order-level distribution and dynamics of APS (potential animal parasites or symbionts) of rice grown in soil of an urban area. (**a**) Order-level distribution of APS in the rhizosphere and phyllosphere. (**b**) Order-level dynamics of APS at seedling, tillering and elongation stages in the rhizosphere and phyllosphere.


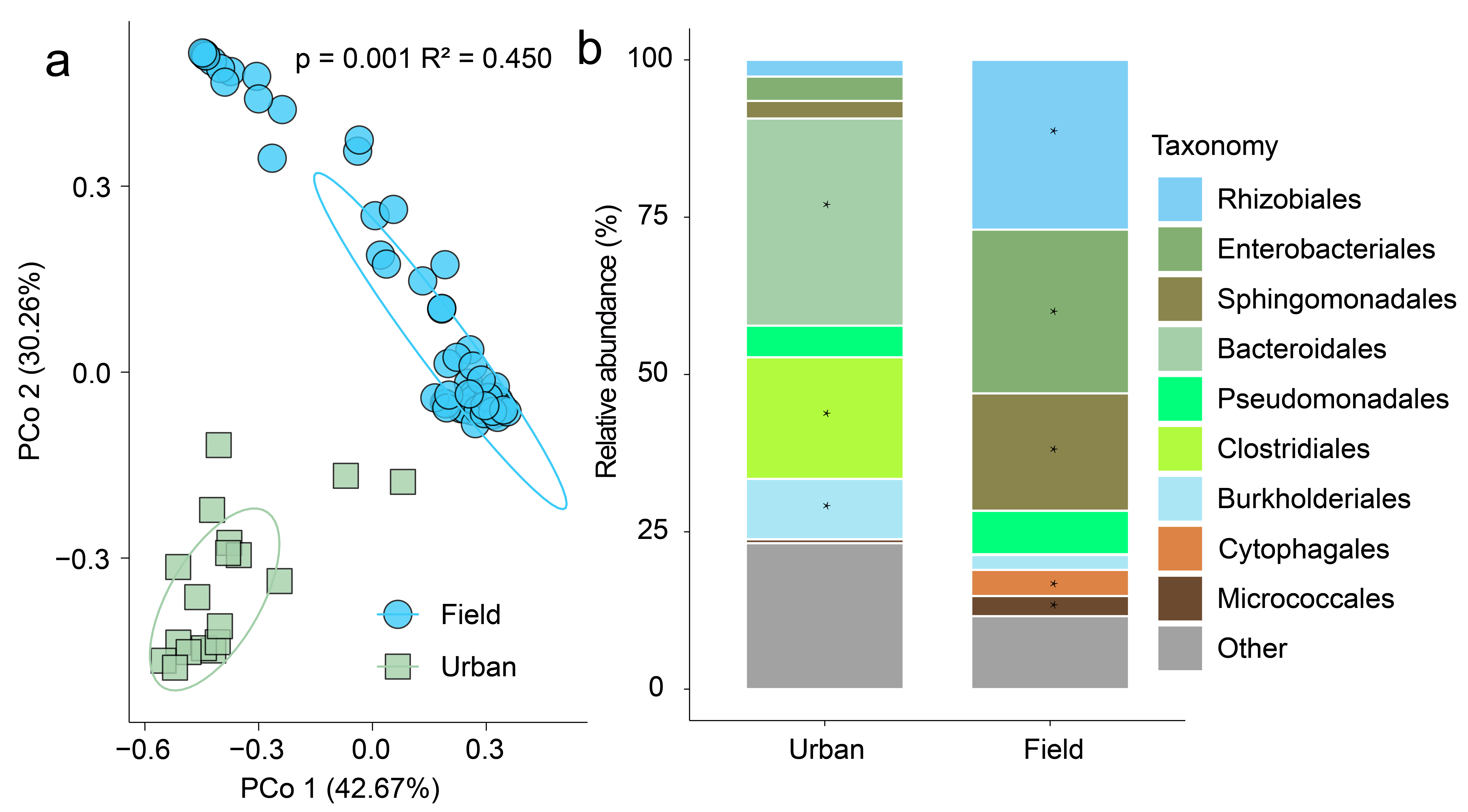


**Supplementary Fig. 9.** Microbiome comparisons for the phyllosphere of rice grown in urban area soil (variety: ZH11) and field (7 varieties: Chakhao, Phouren-mubi, Phoungang, Tolenphou, Moirangphou, Moirangphou khokngangbi, and High Yielding variety). (**a**) Unconstrained PCoA (for principal coordinates PCo1 and PCo2) based on Bray-Curtis distances showing phyllosphere bacterial community clustering of urban area and field (p value was calculated by one-way PERMANOVA). Ellipses cover 68% of the data for each rice subspecies. (**d**) Order-level distribution of bacteria in phyllosphere microbiomes. Asterisks represent significant differences between urban area and field as assessed with unpaired t test (p < 0.05).


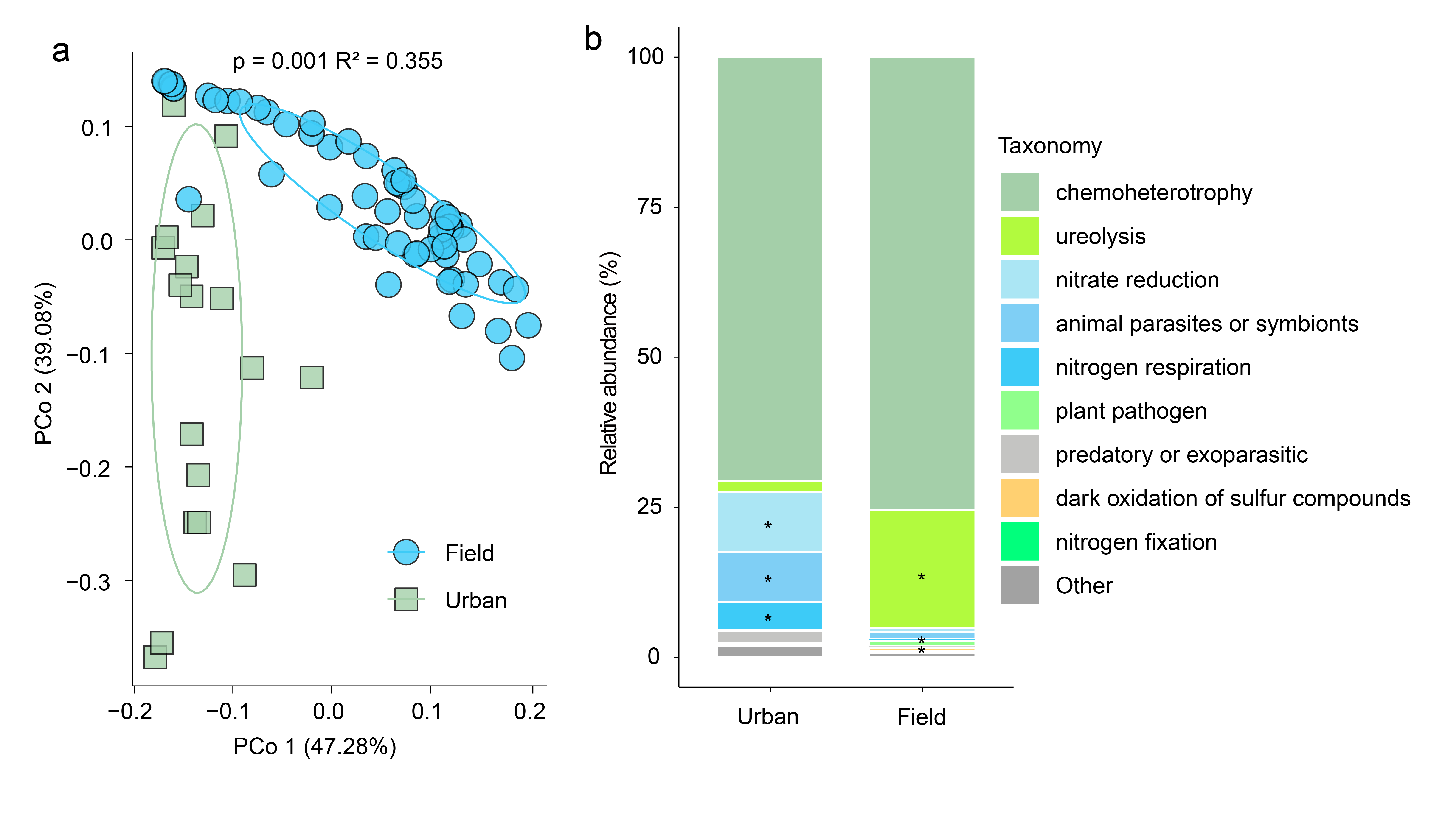


**Supplementary Fig. 10.** Function analysis of the phyllosphere microbiome of rice grown in urban area soil (variety: ZH11) and field (7 varieties: Chakhao, Phouren-mubi, Phoungang, Tolenphou, Moirangphou, Moirangphou khokngangbi, and High Yielding variety). (**a**) Unconstrained PCoA (for principal coordinates PCo1 and PCo2) based on Bray-Curtis distances showing phyllosphere functions of bacterial community clustering of urban area and field (p value was calculated by one-way PERMANOVA). Ellipses cover 68% of the data for each rice subspecies. (**b**) Function distribution of bacteria in the phyllosphere microbiomes of rice grown in urban area soil and a field. Asterisks represent significant differences between urban area and field as assessed with unpaired two sides t-test (p < 0.05).


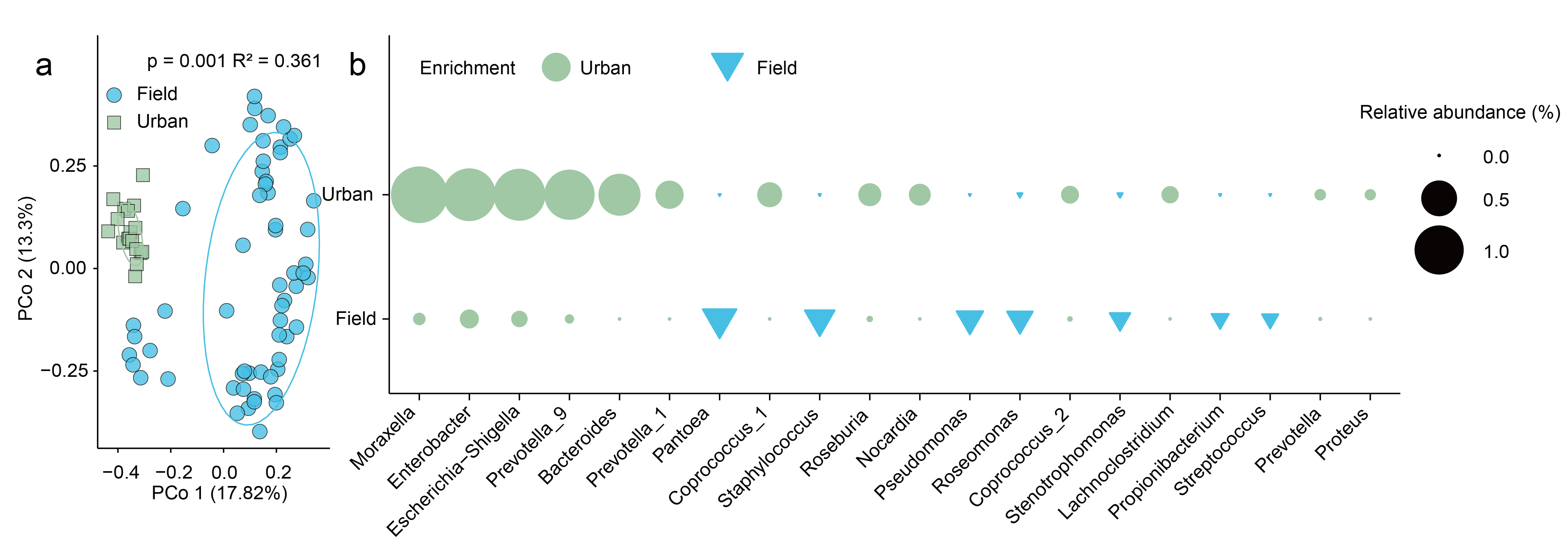


**Supplementary Fig. 11.** Bacteria classified as APS (potential animal parasites or symbionts) inhabiting the phyllosphere of rice grown in soil from an urban area (variety: ZH11) and field (7 varieties: Chakhao, Phouren-mubi, Phoungang, Tolenphou, Moirangphou, Moirangphou khokngangbi, and High Yielding variety). PCoA (**a**) and genus-level distribution (**b**) of APS in the phyllosphere of rice grown in urban area soil and a field.





**Supplementary Fig. 12.** Microbiome comparisons and function analysis of rice grown in urban area soil and field. (**a**) Constrained PCoA (CPCoA) based on Bray-Curtis distances showing bacterial community clustering of urban area and field (p value was calculated by one-way PERMANOVA). (**b**) Order-level distribution of bacteria in microbiomes of rice grown in urban area soil and a field. (**c**) CPCoA showing functions of bacterial community separation for urban area and field (p-value was calculated with PERMANOVA). (**d**) Function distribution of bacteria in the rhizosphere and phyllosphere microbiomes of rice grown in urban area soil and a field. (**e**) CPCoA of, (**f**) relative abundance of, and (**g**) order-level distribution of potential animal parasites or symbionts in the rice grown either in urban area soil or a field. Asterisks represent significant differences as assessed with unpaired two sides t-test (** P < 0.01, *** P < 0.001, **** P < 0.0001). The labels ‘ns’ indicate a not significant difference (P > 0.05). Groups are abbreviated as: phyllosphere of camphor trees, Tree-L; phyllosphere of rice (variety: ZH11), ZH11-L; rhizosphere of rice (variety: ZH11), ZH11-R; phyllosphere of rice (7 varieties: Chakhao, Phouren-mubi, Phoungang, Tolenphou, Moirangphou, Moirangphou khokngangbi, and High Yielding variety), Mix rice-L.

**
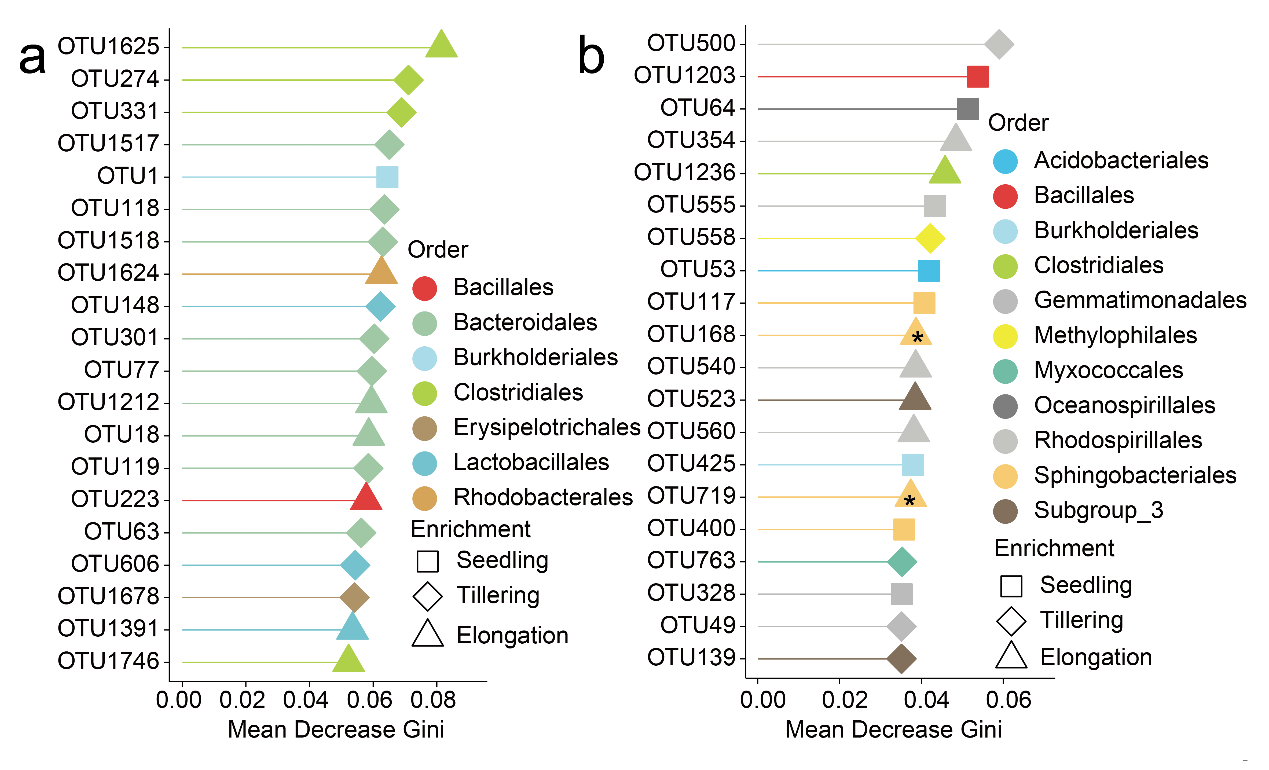
**

**Supplementary Fig. 13.** Specific microbiota members (classified as OTUs) can distinguish developmental stages for (**a**) phyllosphere and (**b**) rhizosphere when after in silico depletion of APS (potential animal parasites or symbionts). The important features are based on Mean Decrease Gini of random forest models. Different shapes represent their enrichment at a certain developmental stages. Different colors indicate the order to which this OTU belongs. Asterisks indicate features that are present in both APS-free and APS-containing microbiomes.


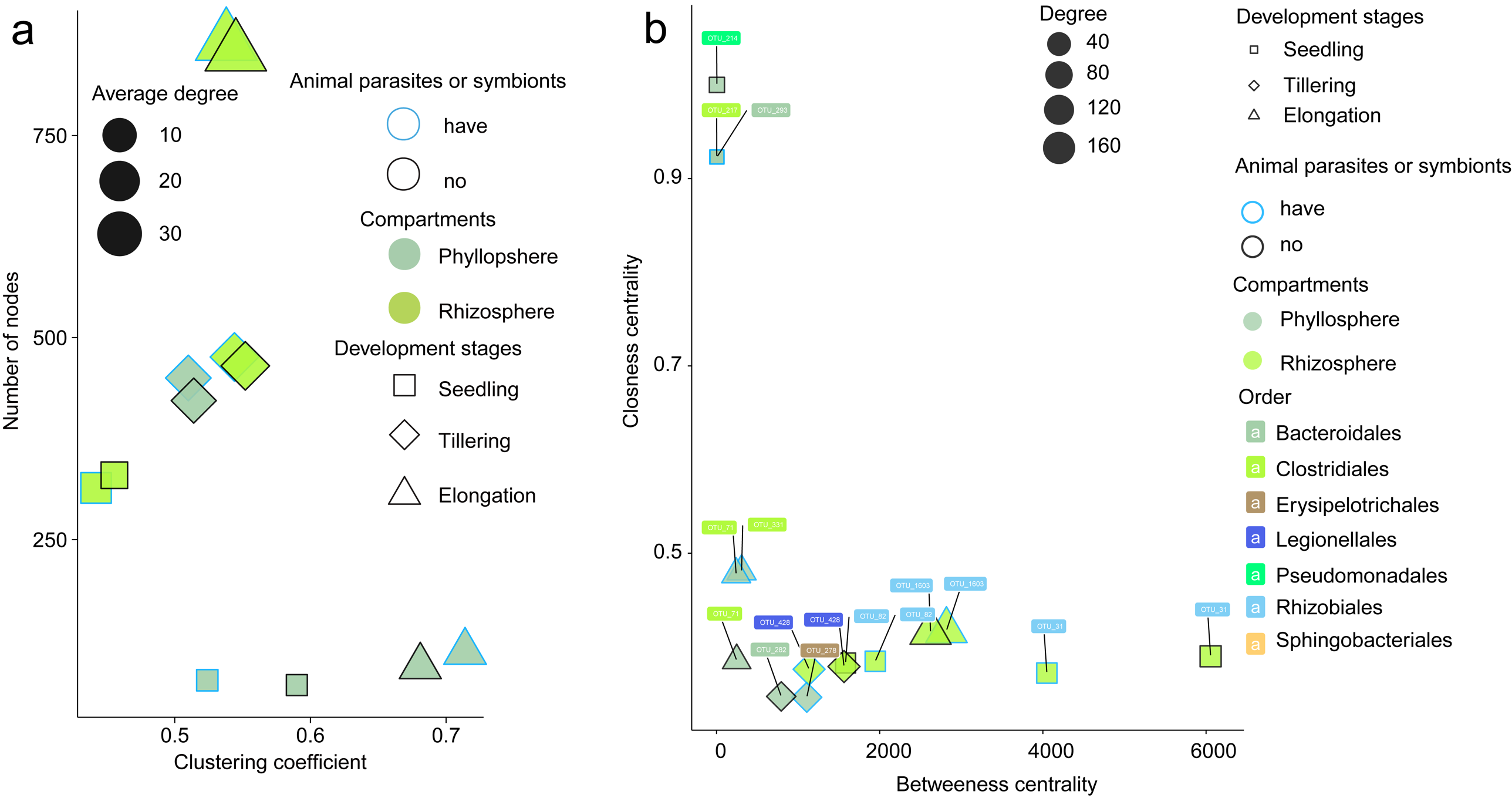


**Supplementary Fig. 14.** Network characteristics (**a**) and hubs (**b**) in APS (potential animal parasites or symbionts)-free and APS-containing microbiome in the phyllosphere and rhizosphere during three development stages.
